# Dissecting Optical Response and Molecular Structure of Fluorescent Proteins With Non-canonical Chromophores

**DOI:** 10.3389/fmolb.2020.00131

**Published:** 2020-07-07

**Authors:** Breland G. Oscar, Liangdong Zhu, Hayati Wolfendeen, Nikita D. Rozanov, Alvin Chang, Kenneth T. Stout, Jason W. Sandwisch, Joseph J. Porter, Ryan A. Mehl, Chong Fang

**Affiliations:** ^1^Department of Chemistry, Oregon State University, Corvallis, OR, United States; ^2^Department of Biochemistry and Biophysics, Oregon State University, Corvallis, OR, United States; ^3^School of Chemical, Biological and Environmental Engineering, Oregon State University, Corvallis, OR, United States

**Keywords:** fluorescent proteins, ultrafast spectroscopy, structural dynamics, vibrational cooling, non-canonical amino acid, femtosecond stimulated Raman

## Abstract

Tracking the structural dynamics of fluorescent protein chromophores holds the key to unlocking the fluorescence mechanisms in real time and enabling rational design principles of these powerful and versatile bioimaging probes. By combining recent chemical biology and ultrafast spectroscopy advances, we prepared the superfolder green fluorescent protein (sfGFP) and its non-canonical amino acid (ncAA) derivatives with a single chlorine, bromine, and nitro substituent at the *ortho* site to the phenolate oxygen of the embedded chromophore, and characterized them using an integrated toolset of femtosecond transient absorption and tunable femtosecond stimulated Raman spectroscopy (FSRS), aided by quantum calculations of the vibrational normal modes. A dominant vibrational cooling time constant of ~4 and 11 ps is revealed in Cl-GFP and Br-GFP, respectively, facilitating a ~30 and 12% increase of the fluorescent quantum yield vs. the parent sfGFP. Similar time constants were also retrieved from the transient absorption spectra, substantiating the correlated electronic and vibrational motions on the intrinsic molecular timescales. Key carbon-halogen stretching motions coupled with phenolate ring motions of the deprotonated chromophores at ca. 908 and 890 cm^−1^ in Cl-GFP and Br-GFP exhibit enhanced activities in the electronic excited state and blue-shift during a distinct vibrational cooling process on the ps timescale. The retrieved structural dynamics change due to targeted site-specific halogenation of the chromophore thus provides an effective means to design new GFP derivatives and enrich the bioimaging probe toolset for life and medical sciences.

## Introduction

Since its discovery several decades ago, green fluorescent protein (GFP) has been widely used for biolabeling and bioimaging due to its characteristic bright green emission, high fluorescence quantum yield (FQY), and stability (Shimomura et al., 1962; Chalfie et al., 1994; Tsien, 1998; Patterson and Lippincott-Schwartz, 2002; Zimmer, 2002; Betzig et al., 2006; Fang et al., 2009; Jung, 2012b; Dedecker et al., 2013). GFP is amenable to structural alterations like circular permutation, leading to the development of biosensors such as the GFP-calmodulin chimera, wherein the fluorescence response is modulated by varying concentrations of free calcium ion (Ca^2+^) (Baird et al., 1999; Zhao et al., 2011; Oscar et al., 2014). Due to the protein utility across broad science and engineering fields, spectroscopic studies have been performed on wild-type (wt)GFP and its derivatives to elucidate the underlying fluorescence mechanisms and predict how further structural changes may alter the optical response including unwanted events such as blinking and photobleaching. On the molecular level, excitation of neutral (A, 395 nm), or anionic (B, 475 nm) absorption bands of the wtGFP autocyclized Ser_65_-Tyr_66_-Gly_67_ (SYG) chromophore results in green fluorescence. After photoexcitation, the neutral chromophore A^*^ undergoes a picosecond (ps) excited-state proton transfer (ESPT) reaction to reach a deprotonated intermediate state (I^*^) within an unrelaxed protein environment preceding green emission (Chattoraj et al., 1996; Lossau et al., 1996; Brejc et al., 1997; Fang et al., 2009).

The optical properties of GFP can be tuned by modifying either the surrounding protein pocket in the β-barrel or the three-residue chromophore (Table S1). For example, point mutation Thr203Tyr of the enhanced yellow fluorescent protein, EYFP, leads to red-shifted absorption and emission due to a π-π interaction between spatially close tyrosine rings (Ormö et al., 1996; Wachter et al., 1998). Since the Tyr sidechain is not mechanistically required for chromophore formation, Tyr66 can be replaced: mutation to His or Trp eliminates the ESPT pathway and shifts the absorption and emission bands to generate blue and cyan fluorescent proteins, respectively (Wachter et al., 1997; Kummer et al., 2002; Ai et al., 2007; Tomosugi et al., 2009). The red fluorescent proteins are typically formed by an extended conjugation along the chromophore N-acylimine carbonyl (Gross et al., 2000; Shaner et al., 2004; Piatkevich et al., 2010; Subach and Verkhusha, 2012). Because these strategies in tuning GFP spectral properties conventionally involve only 20 standard amino acids, they pose certain limitations in achieving desired properties. Notably, the site-specific modification of proteins with non-canonical amino acids (ncAAs) provides an appealing way to engineer spectral properties and encode new functionalities (Link et al., 2003; Wang et al., 2003; Peeler and Mehl, 2012). The GFP chromophore with a *p-*azido-L-phenylalanine mutation, for example, exhibits photoactivatable behavior originating from phenyl azide photolysis in the unnatural chromophore (Reddington et al., 2013). The ncAAs can further act as site-specific vibrational probes or spin labels, making them ideal for structural dynamics techniques such as electron paramagnetic resonance (EPR) spectroscopy, NMR, and time-resolved vibrational spectroscopy (Fleissner et al., 2009; Sripakdeevong et al., 2014; Hall et al., 2019).

In this work, we characterized a series of superfolder GFP (sfGFP) (Pédelacq et al., 2006) mutants that contain a single ncAA point mutation at the chromophore tyrosine residue and compared their attributes to model chromophores using both ultrafast electronic and vibrational spectroscopic signatures (Fang et al., 2018). The halogenated derivatives of sfGFP, 3-chlorotyrosine (Cl-GFP) and 3-bromotyrosine (Br-GFP), contain an electron-withdrawing substituent at the *ortho* site to the phenolic hydroxyl which introduces steric effects in the protein pocket and increases the polarizability of the aromatic bonds over the chromophore ring system. Meanwhile, the 3-nitrotyrosine (nitro-GFP) mutant contains a strong electron-withdrawing group capable of forming additional hydrogen bonds in addition to an ~30 Å^3^ increase in residue volume (De Filippis et al., 2006). Spectral properties such as absorption, emission, and excited-state dynamics are characterized by steady-state and time-resolved electronic spectroscopy; in addition, the chromophore structure and local environment can be revealed by femtosecond stimulated Raman spectroscopy (FSRS) (Dietze and Mathies, 2016; Fang et al., 2019). This integrated experimental platform resolving the coupled electronic and atomic motions in highly fluorescent systems allows us to better understand the effect of a ncAA mutation at the active site, which elucidates the conformational preference of a chromophore inside the protein matrix and the underlying photophysics/photochemistry of fluorescent proteins in the electronic excited states.

## Materials and Methods

### Protein Preparation

The incorporation of a nitrotyrosine (Cooley et al., 2014; Rauch et al., 2016) or halotyrosine (Jang et al., 2020) at a selected position of sfGFP was performed as previously described. Briefly, the codon codifying each of the tyrosine residues in the sfGFP sequence optimized for bacterial expression was replaced by an amber stop codon (TAG), which was recognized by the orthogonal nitro or halotyrosine-bearing suppressor tRNA and engineered tRNA synthetase. The modified proteins were then expressed, purified, and confirmed by mass spectrometry (Jang et al., 2020). The protein concentrations for our ultrafast spectroscopic characterization were 10 mg/mL at pH = 8.1 (10 mM Tris, 50 mM Na_2_HPO_4_, 100 mM NaCl) and 5.5 (100 mM citric acid, 200 mM Na_2_HPO_4_, 100 mM NaCl). As control samples, the monohalogenated 4-hydroxybenzylidene-1,2-dimethylimidazolinone (HBDI) chromophores were synthesized according to literature by combining an iminoglycine methyl ester with a Schiff base (Baldridge et al., 2010), as detailed in the Supplementary Text.

### Spectroscopic Methods

The UV/Visible and emission spectra of all proteins and small molecules were collected on a ThermoScientific Evolution 201 and Hitachi F-2500 fluorescence spectrophotometer, respectively. Quantum yield was measured relative to fluorescein in 0.1 M NaOH according to the reported method (Patterson et al., 1997). The tunable picosecond (ps) Raman pump, white light probe, and femtosecond (fs) actinic pump (see Supplementary Text) (Zhu et al., 2014; Liu et al., 2016) enable the acquisition of time-resolved FSRS data in the electronic excited state with simultaneously high spectral and time resolutions (Dietze and Mathies, 2016; Fang et al., 2018). Transient absorption (TA) spectra were collected before each experiment with the Raman pump blocked so that only the fs pulses interact with the sample. The UV/Visible spectra were recorded before and after time-resolved experiments to check sample integrity (<5% change commonly detected). A full description of the methods can be found in our earlier reports on fluorescent proteins (Tang et al., 2016, 2018a; Fang et al., 2018).

## Results and Discussion

### Steady-State Electronic Spectroscopy

**Figure 1:**
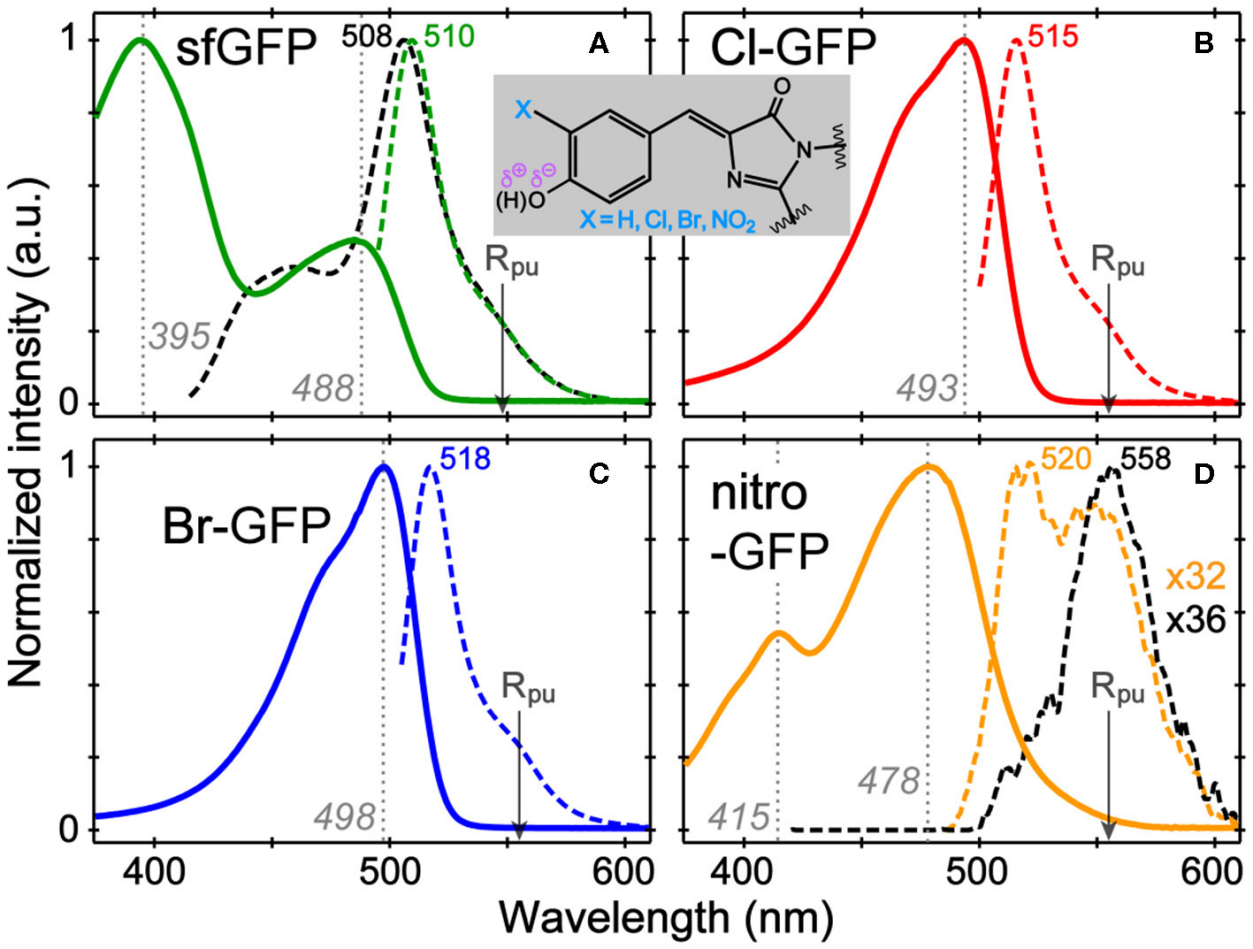
Normalized absorption (solid) and emission (dashed lines) spectra of **(A)** sfGFP, **(B)** Cl-GFP, **(C)** Br-GFP, and **(D)** nitro-GFP in pH = 5.5 aqueous buffer solution. The excitation wavelengths are marked by gray dotted lines. For sfGFP and nitro-GFP, emission after the bluer excitation is shown as black dashed lines. The nitro-GFP emission normalization factor is denoted to manifest weak emission. The Raman pump is indicated by the arrow. The chromophore chemical structure is shown in the inset.

The absorption and emission spectra of sfGFP, Cl-GFP, Br-GFP, and nitro-GFP in pH = 5.5 buffer solution (Figure 1) are tabulated in Table S2. The p*K*_a_ of sfGFP is ~6 so a distinct neutral chromophore population (λ_max_ = 395 nm) is observed (Figure 1A), whereas in more alkaline conditions the deprotonated chromophore population (λ_max_ = 488 nm) dominates (Figure S1A). We observed a lowered p*K*_a_ of ~4.5 upon tyrosine halogenation inside sfGFP, so the neutral chromophore absorption around 400 nm is largely absent at pH = 5.5 while the anionic chromophore absorption bands red-shift (Figures S1B,C). These results are corroborated by the p*K*_a_ of 3Cl-Tyr EGFP at ~4.7 and red-shifted spectral peaks vs. EGFP (Ayyadurai et al., 2012; Zhang et al., 2012). The emission profiles of Cl-GFP and Br-GFP are also red-shifted vs. sfGFP, and the shift magnitude increases with the mass of the halogen substituent (Figures 1A–C), the trend matching 3-iodotyrosine-GFP with a red-shifted emission beyond 520 nm (Young et al., 2011). Notably, changes in absorption and emission for chromophores in a protein matrix are more pronounced than those in small molecules. The absorption bands of the sfGFP model chromophore, neutral, and anionic *p-*HBDI with capped methyl groups, are found at 370 and 425 nm, respectively (Vengris et al., 2004; Taylor et al., 2019). The corresponding Cl (Br)-HBDI bands appear at 369 (368) nm and 424 (425) nm in pH = 3 and 7.6 aqueous solutions, respectively.

In contrast, the nitrated GFP chromophore results in a non-fluorescent protein, which is not surprising given the photochemistry of nitrated aromatics (see Supplementary Text for the mechanism; De Filippis et al., 2006; Tang and Fang, 2019). The absorption spectrum shows two bands at 415 and 478 nm that exhibit a pH-dependent ratio change with the 415 nm peak becoming stronger under acidic conditions (Figure S1D). The 415 nm band likely corresponds to neutral chromophore in nitro-GFP and is red-shifted from the 395 nm absorption band in sfGFP, which could be due to an intramolecularly H-bonded form in nitro-GFP. Excitation of either absorption band produces negligible fluorescence (Φ < 0.0005, see Table S2) and the detectable emission is significantly red-shifted to ~550 nm. This behavior is reminiscent of free 3-nitrotyrosine in solution, which shows the pH-dependent changes of its visible absorption bands, a p*K*_a_ near neutral pH, and an FQY below 0.0001 (Tang and Fang, 2019), leading to the effective use of 3-nitrotyrosine as a FRET quencher in peptides and proteins when a nearby fluorescent residue (e.g., Tyr, Trp) acts as the donor (Duus et al., 1998; De Filippis et al., 2006).

### Stimulated Raman Spectroscopy in the Electronic Ground and Excited States

To verify the chromophore's ionization state and uncover local interactions within the protein pocket, we implemented the wavelength-tunable FSRS technique at different resonance conditions (Liu et al., 2016; Fang et al., 2018), wherein a narrowband Raman pump and broadband Raman probe induce the stimulated Raman scattering signal with desirable enhancement to achieve high signal-to-noise ratio. The ncAA chromophores exhibit unique spectral signatures when compared to sfGFP; for example, the halogenated chromophores contain highly polarizable groups that affect Raman peak frequencies, and the nitrated chromophore consists of the spectrally isolated –NO_2_ vibrational modes that act as sensitive probes for the local environment (De Filippis et al., 2006; Ayyadurai et al., 2012).

**Figure 2:**
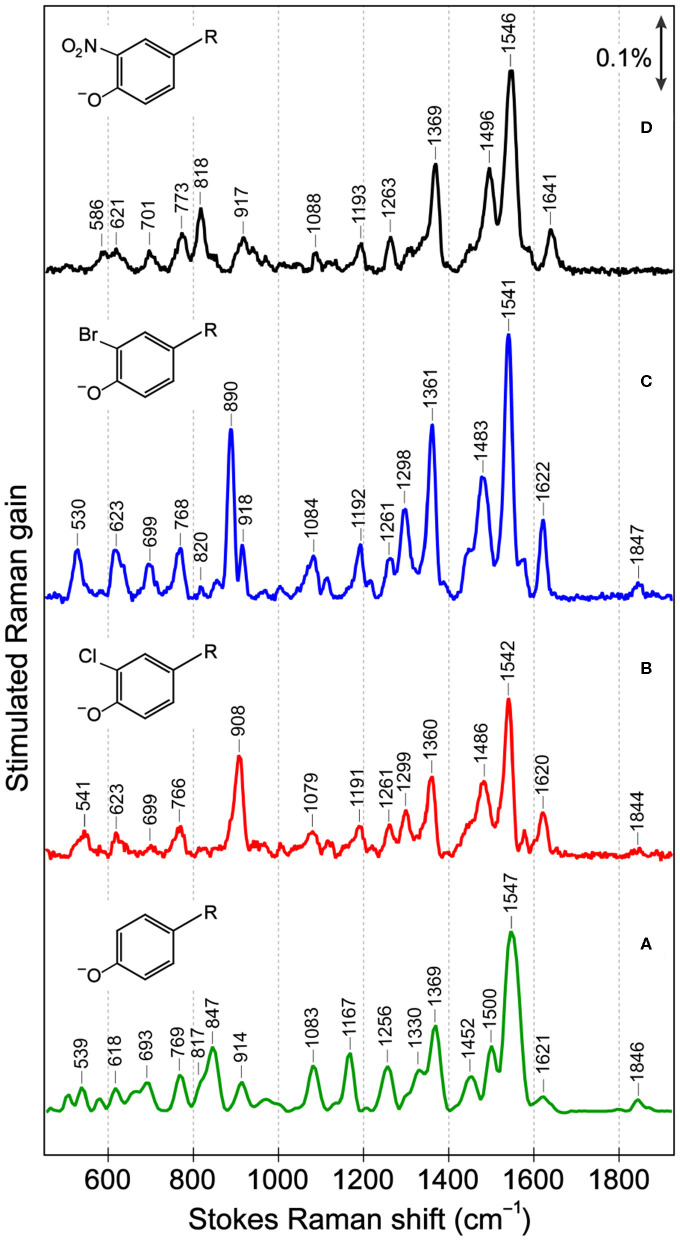
Ground-state Stokes FSRS data of **(A)** sfGFP, **(B)** Cl-GFP, **(C)** Br-GFP, and **(D)** nitro-GFP in pH = 5.5 aqueous buffer solution. In **(B–D)** the Raman pump was set at 555 nm while in **(A)** the Raman pump wavelength was 548 nm. The stimulated Raman gain is indicated by the double-headed arrow. The anionic chromophore chemical structures with various substitutions are depicted in the inset. *R* represents the remaining conjugated framework of the chromophore that connects to protein backbone.

In the ground-state FSRS of the protein series in pH = 5.5 buffer (Figure 2), the Raman pump is energetically close to the absorption band of the anionic chromophore (Figure 1). In sfGFP, most of the Thr-Tyr-Gly (TYG) chromophore population is neutral, but the pre-resonance condition favors the anionic subpopulation (see Figure S1A) and amplifies its Raman features. The protonation state is confirmed by the 1,547 cm^−1^ marker band, attributed to the C=N, C=C, and C=O stretching motions in the anionic chromophore (see Table S3 for vibrational normal mode assignments) based on literature and our calculations (Bell et al., 2000; Schellenberg et al., 2001; Tozzini and Nifosì, 2001). For proteins with primarily neutral chromophores, this marker band shifts to ~1,566 cm^−1^, also observed in wtGFP (Fang et al., 2009), a series of GFP-based Ca^2+^ biosensors (Oscar et al., 2014; Tang et al., 2016), and in sfGFP at an off-resonance condition (Figure S2A). The ncAA-mutant proteins (Figures 2A–C, also see Figure S2B for the off-resonance FSRS data of Br-GFP) all exhibit strong peaks near 1,542 cm^−1^, corroborating the anionic chromophore as determined by the electronic absorption spectra (Figure 1). The ~1,576 cm^−1^ shoulder peak in mutant proteins is assigned to additional phenolate ring C=O and C=C stretch contributions in the anionic chromophore (Tables S4, S5), but this mode may indicate an H-bonded population of halogenated chromophores while the H-bond partner could be an adjacent water or protein residue in forming the O–H···X (X = Cl, Br) bond (Pal et al., 2005). In the anionic chromophores outside the protein matrix, strong C=C and C=O stretching modes appear at 1,560 cm^−1^ (Cl-HBDI) and 1,558 cm^−1^ (Br-HBDI) in Figure S3, slightly blue-shifted from the reported 1,556 cm^−1^ mode of HBDI in basic solution (Bell et al., 2000; Schellenberg et al., 2001; Taylor et al., 2019).

The cluster of modes between ~1,200 and 1,400 cm^−1^ also probe the chromophore's protonation state: the 1,256 cm^−1^ mode in sfGFP involves the phenolate ring H-rock and CO stretch, which typically exhibits a frequency blueshift in the deprotonated state (Bell et al., 2000; Fang et al., 2009; Oscar et al., 2014). This mode blue-shifts to 1,261 cm^−1^ in Cl-GFP and Br-GFP, consistent with the incorporation of an electron-withdrawing group adjacent to the phenolate oxygen site and the increased acidity as well as photoacidity of the chromophore (Chen et al., 2019). In contrast, for the phenolate ring H-rocking and imidazolinone ring C–N stretching mode at 1,369 cm^−1^ (Table S3) that was also observed for the TYG chromophore inside a protein Ca^2+^-biosensor (Tang et al., 2016), due to steric hindrance this mode red-shifts to ~1,360 cm^−1^ upon chromophore halogenation (Figure 2). Moreover, the 1,167 cm^−1^ phenolate ring H-scissoring motion exhibits a notable blueshift to ~1,192 cm^−1^ in Cl-GFP and Br-GFP since the pertinent normal modes of the halogenated chromophores involve less imidazolinone ring contributions (Tables S3–S5). These in-plane vibrational motions thus serve as sensitive probes to elucidate the effect of *ortho*-halogenation of the largely planar chromophore inside a protein pocket.

**Figure 3:**
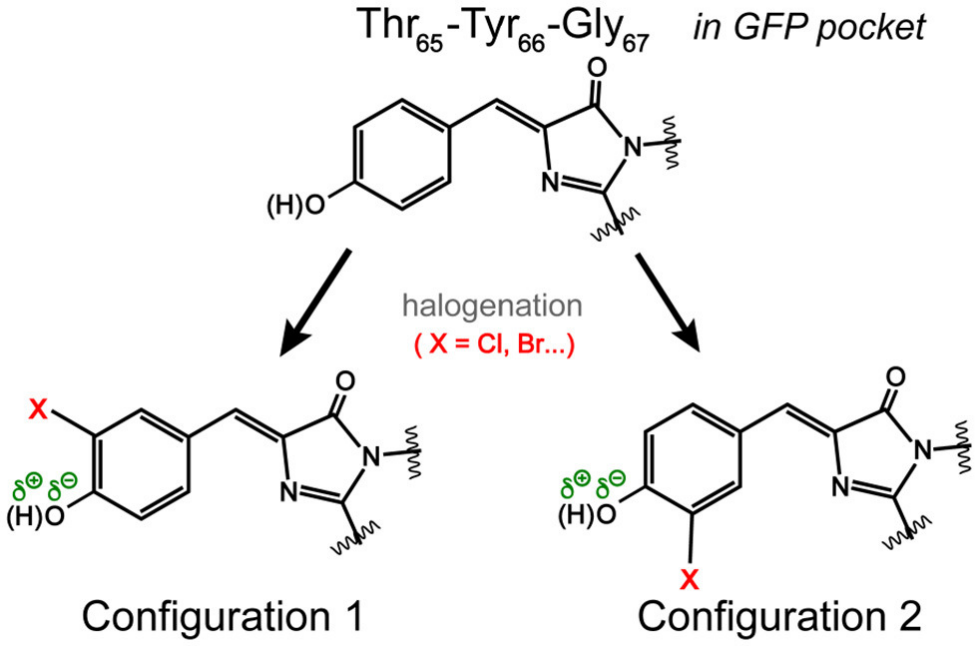
Possible orientations of the halogen atom (X) on the GFP chromophore. In Configuration 1, the halogen atom is oriented toward Thr203 in a typical GFP protein matrix. In Configuration 2, the halogen atom could face Ser205 (Pal et al., 2005). The dissociable hydroxyl group is highlighted.

Notably, the chromophore autocyclization during protein maturation is primarily a function of the protein backbone. A majority of GFP chromophores are observed in the *Z* (*cis*) stereoisomer, but the halogen substituent on tyrosine can occupy two distinct atropisomeric positions with the probability of each determined by the properties of the substituent itself as well as the local environment supplied by the protein interior (Bae et al., 2004; Pal et al., 2005; Jung, 2012a; Chang et al., 2019). For example, the crystal structure and electron density mapping of 3-fluorotyrosyl-EGFP revealed two conformations of the TYG chromophore with a major conformer wherein fluorine interacts with Thr203, equivalent to Configuration 1 in Figure 3 (Bae et al., 2004). Small-molecule analogs of the chromophore were reported with this conformation as well as the Trp-containing chromophores and the 3,4-dihydroxy-L-phenylalanine GFP chromophore (Hyun Bae et al., 2003; Hasegawa et al., 2007; Ayyadurai et al., 2011). However, 3-chlorotyrosine chromophores in the short H-bond (His148Asp) GFP system exhibit only one crystallographic occupancy corresponding to Configuration 2 in Figure 3 partly due to specific electrostatic interactions introduced by the nearby His148Asp mutation (Oltrogge and Boxer, 2015). Can FSRS provide evidence for the protein chromophore configuration?

Several low-frequency motions below 1,000 cm^−1^ are resolved for sfGFP and the mutated series (Figure 2), and these modes are sensitive to the chromophore conformation. The C–Cl stretch (550–800 cm^−1^), C–Br stretch (500–700 cm^−1^), and NO_2_ bend (~820 cm^−1^) are all expected to occur in this region (Kovács et al., 1998; Chen et al., 2020). Mode assignment of the anionic TYG chromophore and the halogenated derivatives in both conformations (Figure 3) was carried out by comparing the experimental Raman spectra to literature values and density functional theory (DFT) RB3LYP-level calculations with the 6-311G+(d, p) basis set performed *in vacuo* and in water. Strong vibrational modes at 908 cm^−1^ in Cl-GFP and 890 cm^−1^ in Br-GFP are assigned to phenolate ring breathing with a prominent C–X stretching component. The ring breathing mode has been previously assigned at ~820 cm^−1^ (Oscar et al., 2014) that is also listed in Table S3, but the C–X stretching contribution blue-shifts the calculated normal mode frequency (see Tables S4, S5). Similarly, the benzene ring breathing mode at 992 cm^−1^ was experimentally recorded at 1,001 cm^−1^ in chlorobenzene and 998 cm^−1^ in bromobenzene, so this mode is a useful reporter on halogen substitution (Meneely et al., 1971).

**Figure 4:**
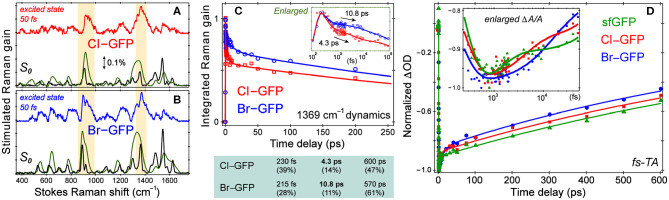
Spectroscopic characterization of ncAA-GFPs. Ground and excited-state Stokes FSRS of **(A)** Cl-GFP and **(B)** Br-GFP. The spectra with the 555 and 507 nm Raman pump are depicted in black and green (×0.05), respectively. The excited-state Raman spectrum at 50 fs after 480 nm photoexcitation is shown in red (Cl-GFP) and blue (Br-GFP). The stimulated Raman gain of 0.1% is indicated by the double-headed arrow. **(C)** Normalized Raman intensity dynamics of the 1,369 cm^−1^ band of Cl-GFP (red) and Br-GFP (blue) with the least-squares fit in solid lines. Early-time dynamics are highlighted in the inset on a semilogarithmic scale. The triexponential-fit components are listed below with the amplitude weight percentages of decay in parenthesis. **(D)** Fs-TA dynamics of the SE band (550−570 nm) of sfGFP (green), Cl-GFP (red), and Br-GFP (blue) in pH = 5.5 aqueous buffer solution following 480 nm excitation. The data are normalized at the maximal SE peak magnitude point for comparison. The least-squares fit for each data trace is shown as the color-coded solid curve. The inset shows the early-time dynamics on a semilogarithmic scale to highlight the multiple timescales involved.

Resonance Raman spectra with a Raman pump close to the red edge of electronic absorption bands (Figure 1, Figure S1) were used to further assign the low-frequency vibrational bands (Figures 4A,B, Figure S4), supported by the anti-Stokes FSRS with a 580 nm Raman pump (Figure S5) (Tang et al., 2018b). With identical protein concentration and Raman pump power, the signal strength is increased by an order of magnitude with 507 nm pump. Resonance Raman peaks include excited-state contributions (Quick et al., 2015), which can be seen by strong agreement between the resonance Raman spectra and the excited-state spectra at 50 fs following 480 nm photoexcitation. Since halogen atoms are expected to increase chromophore polarizability, the modes strongly influenced by halogenation are enhanced (see Supplementary Text). The lineshape of high-frequency modes is largely preserved in all proteins, though the peaks in S_1_ are generally broader than those in S_0_ due to the shorter lifetime of the excited-state species, while the frequency shift is due to an interplay between anharmonicity and the intrinsic frequency change from S_0_→*S*_1_ due to electron redistribution (Chen et al., 2018; Fang et al., 2018).

Based on the ground-state FSRS and calculations, we tentatively assign Br-GFP to Configuration 1 in Figure 3 such that bromine interacts with the nearby Thr203 residue. Though DFT calculations of the model chromophore *in vacuo* cannot capture the myriad of interactions between the protein pocket and the chromophore, there is better agreement between the experimental and calculated vibrational modes of Configuration 1 of Br-GFP, especially in the low-frequency region (Merrick et al., 2007; Wang et al., 2015). In particular, the observed 890 and 918 cm^−1^ modes (Figure 2C) both have significant Br contributions and the calculated frequencies of 852 and 881 cm^−1^ in Configuration 1 (Table S5) better match the experimental energy gap of 28 cm^−1^ between the modes, instead of the exact mode frequencies that are highly subjective to the frequency scaling factor (Merrick et al., 2007). A crystallographic analysis is required to confirm if a minor population exists; however, we expect the bromine to experience more repulsive interactions with the sidechains of Ser205 and Glu222 in Configuration 2 based on the sfGFP crystal structure (PDB ID: 2B3P) (Pédelacq et al., 2006). Our preliminary molecular dynamics simulations based on free energy perturbation methods (Seeliger and de Groot, 2010) with a calculated chromophore force field (Malde et al., 2011), however, seem to suggest that while both configurations are stable in the protein pocket, Configuration 2 of Cl-GFP is thermodynamically more favorable. Further investigation is thus needed to better determine which configuration is dominant surrounded by dynamic protein residues in the system of interest.

### Excited-State Electronic and Structural Dynamics of the Halogenated sfGFP

While the small molecule analog HBDI undergoes a non-radiative *cis*-to-*trans* isomerization after photoexcitation (Mandal et al., 2004; Taylor et al., 2019), confinement in the protein pocket typically inhibits this pathway in favor of other energy dissipation routes. To rule out photoisomerization in the excited state and explore the photodynamics affected by halogenation, we implemented time-resolved electronic and vibrational spectroscopies (Liu et al., 2016; Fang et al., 2019). Using an fs photoexcitation pulse at 480 nm and a white light probe, TA spectra were collected to reveal dynamics in the first singlet excited state. The sfGFP, Cl-GFP, and Br-GFP all have a broad stimulated emission (SE) feature below 600 nm (in correlation with the fluorescence band in Figure 1, Figure S1) and a weak excited-state absorption band beyond 600 nm (Tang et al., 2015). We focus on TA dynamics by plotting the red-edge integrated signal of SE band from 550–570 nm, which rapidly reaches the maximum magnitude before a biexponential decay (see Figure 4D, Table S6). For sfGFP in pH = 5.5 buffer, only a small portion of the chromophore population is excited at 480 nm, and a ~1.2 ps component accounts for 20% of the SE dynamics while a longer 1.2 nanosecond (ns) component is dominant. A similar time constant of 1.9 ps for the initial rise of fluorescence signal was reported in the deprotonated chromophore of GFP after 478 nm excitation (Chattoraj et al., 1996), likely arising from an ultrafast process (e.g., intramolecular vibrational relaxation; Felker and Zewail, 1985) that populates the fluorescent state of the deprotonated species. The long time component is less accurately determined due to the 600 ps detection window (see **Section 2.2**), but generally approaches the fluorescence lifetime (~3 ns) of the photoexcited deprotonated chromophore (Chattoraj et al., 1996; Striker et al., 1999; Zimmer, 2002; Tang et al., 2018c).

Notably, the first recovery component of the SE band of halogenated proteins is significantly longer than the parent protein (see Figure 4D inset and Table S6), but the ns process is largely unchanged. The 4.1 ps component in Cl-GFP lengthens to 12.4 ps in Br-GFP, and these time constants are attributable to the excited state (S_1_) relaxation dynamics other than fluorescence (*vide infra*), especially with the excess energy provided by the 480 nm pump. The excellent match between these TA time constants and the aforementioned Raman mode intensity decay time constants (Figure 4C, lower panel) supports a unified picture for energy relaxation on molecular timescales of a photoexcited deprotonated chromophore inside the protein pocket (Tang et al., 2018c; Fang et al., 2019). Previous photoelectron spectroscopic results of the UV-irradiated anionic HBDI chromophore (Mooney et al., 2013) and that with chemical modifications (e.g., difluoro-substituents) (Bochenkova et al., 2017) corroborate the changes in the excited-state energy surfaces and variation of the 1.4 ps lifetime (in gas phase and solution) following an initial ~330 fs component. Recently with time-resolved action spectroscopy, the HBDI anion after 480 nm pump shows ca. 1–11 ps lifetimes at 300 K (Svendsen et al., 2017).

To verify that the observed dynamics arise from a vibrational progression, we performed the time-resolved FSRS experiments in S_1_ to directly track atomic motions (Fang et al., 2018). In previous FSRS reports on GFP derivatives, the modes with strong intensities are typically the phenol C–H bending motions, phenolic CO stretch, and the imidazolinone C=N stretch with frequencies at ~1,180, 1,265, and 1,565 cm^−1^ in S_1_ (Fang et al., 2009; Oscar et al., 2014; Tang et al., 2015, 2016). These high-frequency marker bands are also prominent in the pre-resonance ground-state Raman spectrum of sfGFP (Figure S4). Interestingly, halogenation changes this pattern by exhibiting several enhanced low-frequency modes. In the excited state, modes with major C–X contributions exhibit the strongest intensities, and the mode frequencies are blue-shifted from those in S_0_ (Figures 4A,B). Following 480 nm photoexcitation, the S_1_ vibrational modes of Cl-GFP and Br-GFP decay in time without the appearance of new peaks, while only small mode frequency blueshift occurs (Figure S6). The ~1,369 cm^−1^ mode is present in S_0_ and S_1_ spectra of halogenated proteins and could be the vibronically coupled mode based on the energy gap observed in the electronic spectra (see Supplementary Text and Figure 1). This marker band dynamics are fit with a triexponential function (Figure 4C), largely matching the fs-TA dynamics (Figure 4D). Notably, Cl-GFP exhibits a ~4 ps decay time constant that is much shorter than the ~11 ps counterpart of Br-GFP, in correlation with the noticeable mode frequency blueshift in Figure S6 that indicates vibrational cooling in S_1_ (Fang et al., 2019).

Interestingly, the amplitude weights of the initial ps components (i.e., 20, 37, and 27% in Table S6) correlate with the FQYs (i.e., 0.68, 0.88, and 0.76 in Table S2) of sfGFP, Cl-GFP, and Br-GFP, respectively. We surmise that the 4–12 ps components in Cl-GFP and Br-GFP (longer than 1.2 ps in sfGFP) involve certain nuclear motions associated with the phenolate ring as its size/weight increases, which allow effective vibrational cooling that promotes radiative emission from the lower portion of the potential energy surface of the deprotonated protein chromophore (Fang et al., 2018; Tang et al., 2018c). This mechanism is corroborated by a recent report on the introduction of asymmetric electronic structures and vibronic features to fluorophores, which can facilitate strong internal conversion with redder emission (Ren et al., 2018). Notably, photoisomerization typically leads to characteristic Raman mode frequency redshift due to the chromophore conformational change (Fang et al., 2019), which was not observed here (Figure S6). Moreover, the ring-twisting-induced non-radiative transition contradicts the high FQYs of halogenated sfGFP (Table S2), whereas the essentially non-fluorescent nitro-GFP likely involves an ultrafast nitroaromatic twisting motion leading to an S_1_/S_0_ conical intersection (Tang and Fang, 2019). One challenge that needs to be tackled before future FSRS experiments on nitro-GFP is the low signal-to-noise ratio without a prominent SE band (see Supplementary Text for details) like that in the halogenated sfGFP achieving resonance Raman enhancement in S_1_ (Figure S6).

## Conclusions

In summary, we prepared and characterized a series of superfolder GFP mutants with ncAA chromophores using a combination of fs-TA spectroscopy, wavelength-tunable ground and excited-state FSRS (with ncAA chromophores in solution as control samples), and DFT calculations of normal mode frequencies. In particular, the single-site halogenated proteins display improved properties that include the red-shifted absorption and emission, increased concentration of deprotonated emissive species, and an increased fluorescence quantum yield. Such desirable application properties of the halogenated GFP mutants stem from a solid biophysical chemistry foundation in that they are a direct consequence of the engineerable molecular structure and dynamics of the photosensitive unit inside a protein matrix. The nitro-GFP provides a useful contrasting sample that will be further investigated.

We focused on the structural aspects of single-site halogenation at the protein active site to examine key conformational preference and elucidate the excited-state energy dissipation pathways in Cl-GFP and Br-GFP. Such a targeted analysis using a well-known series of electron-withdrawing groups with sufficient temporal and spectral resolution paints a more complete picture of chemically modified chromophores reacting to the incoming photons, thus enabling future rational design of functional molecular machines (Fang et al., 2019). The strong vibronic coupling that influences the SE dynamics may provide a useful direction to engineer probes for SE-depletion spectroscopy and imaging (Hell, 2009; Silva et al., 2016). Furthermore, these brighter protein mutants show that ncAA incorporation within the chromophore is a versatile and effective way to engineer photochemistry and protein functionality.

## Data Availability Statement

The raw data supporting the conclusions of this article will be made available by the authors, without undue reservation.

## Author Contributions

RM and CF conceived and designed the research and acquired funding. BO and LZ performed spectroscopic experiments. BO, NR, AC, and JS performed data curation including calculations. HW, JP, and KS contributed new protein and chromophore samples. LZ and CF contributed advanced non-linear spectroscopic tools. BO and CF wrote the manuscript. All the authors have edited the final manuscript and approved it for publication.

## Conflict of Interest

The authors declare that the research was conducted in the absence of any commercial or financial relationships that could be construed as a potential conflict of interest.
