# Supplementary material for: Dissecting Optical Response and Molecular Structure of Fluorescent Proteins With Non-canonical Chromophores
Source: Front Mol Biosci. 2020 Jul 7;7:131. doi: 10.3389/fmolb.2020.00131 (PMC7358599; doi:10.3389/fmolb.2020.00131)
Supplement: Supplementary file 1 [file Data_Sheet_1.PDF]

# Supplementary Material

## 1 Supplementary Text

**GFP chromophore synthesis methods.** To prepare the halogenated model chromophores as control samples for the study of halogenated sfGFP derivatives (see main text), the Schiff bases were synthesized by the condensation of either 3-chloro-4-hydroxybenzaldehyde (**1**) or 3-bromo-4-hydroxybenzaldehyde (**2**) purchased from TCI America and used without further purification with 30% wt methylamine (**3**) in ethanol in a 1:1.1 molar ratio. The reaction between **1** and **3** went overnight while the reaction between **2** and **3** proceeded for 48 hours. Ethyl acetimidate hydrochloride and glycine methyl ester hydrochloride were combined with potassium carbonate, water, and diethyl ether to produce 1-ethoxyethylideneamino acetate, which was used to react with the halogenated Schiff base for 18 hours. The final products were crystallized in ethanol and dried overnight. A plug of silica gel column was run with a 2:1 dichloromethane to acetone solvent mixture and the yellow solid products recrystallized. $^{1}$H and $^{13}$C NMR were performed to confirm the synthesized product (Kojima et al., 1998; Chen et al., 2019; Taylor et al., 2019), which was dissolved in solution as control samples.

**Tunable femtosecond stimulated Raman spectroscopy (FSRS) methods.** To generate the wavelength-tunable picosecond (ps) Raman pump at 550 nm, the fundamental output (800 nm) from a Ti:sapphire regenerative laser amplifier was passed through a home-built system that consists of a femtosecond (fs) noncollinear optical parametric amplifier (NOPA), a spectral filter, and a two-stage ps NOPA pumped by the 400 nm ps pulse out of a second harmonic bandwidth compressor (SHBC) (Zhu et al., 2014; Liu et al., 2016). The broadband Raman probe was generated as supercontinuum white light from a 2-mm-thick sapphire crystal plate. To collect the ground-state FSRS, the Raman pump (average power of 3–4 mW) was overlapped with Raman probe on 1-mm-pathlength quartz cuvette with a micro stir bar, constantly moved by an external magnet to ensure the fresh sample is irradiated by each laser pulse. The fs 480 nm photoexcitation pulse was converted from the 800 nm fundamental pulse via a two-stage fs NOPA and reduced to ~0.3 mW that showed a good balance between the observed FSRS signal intensity and protein sample integrity in aqueous buffer solution (Tang et al., 2018a; Tang et al., 2018b). In general, the stimulated Raman signal is much stronger than the continuous-wave spontaneous Raman signal so we can capitalize on resonance conditions and further enhance the signal-to-noise ratio of the FSRS signal (Fang et al., 2018; Fang and Tang, 2020). To check the photostability of proteins in buffer solution, we routinely measure the UV/Visible spectra of the protein samples before and after the time-resolved spectroscopic experiments (e.g., fs transient absorption and FSRS), and a typical absorption spectral change within 5% of the original values shows that the proteins under study are stable under the implemented moderate laser irradiation conditions (Fang et al., 2009; Tang et al., 2015; Fang and Tang, 2020). Alternatively, the fluorescence spectrum can be used as a spectroscopic check for the protein photostability. The laser excitation power (e.g., for the 480 nm actinic pump used in **Figure 4**) can be adjusted in transient absorption and FSRS experiments to achieve sufficient signal-to-noise ratios while maintaining the integrity of proteins in buffer solution (Fang and Tang, 2020).

**Additional discussions of the FSRS spectra of halogenated sfGFP derivatives.** The ground-state triplet peaks between 1200–1400 cm$^{-1}$ of Cl-GFP and Br-GFP (see **Figure 2b-c** with a 555 nm Raman pump in main text) are significantly enhanced and broadened with a 507 nm Raman pump (**Figure 4a-**

**b**), though the strongest contribution to resonance enhancement seems to be the ~1300 $cm^{-1}$ mode. These modes involve the phenolate ring, bridge, and imidazolinone deformations (**Table S4-S5**), which are expected to be enhanced in the excited state as electron density is transferred from the phenolate ring to the imidazolinone ring, driven by the chromophore photoacidity in these GFP systems (Fang et al., 2009; Fang et al., 2019). Moreover, the intensity ratio between the 890 $cm^{-1}$ phenolate ring breathing and C–Br stretching mode and the neighboring 918 $cm^{-1}$ phenolate ring and bridge H-out-of-plane (HOOP) mode is 3.15 (**Figure 2c**), but this ratio drops to 1.34 in the resonance Raman spectrum (**Figure 4b**). Interestingly, the corresponding Cl-GFP mode at 917 $cm^{-1}$ in the resonance Raman spectrum is rather broad (**Figure 4a**) and blue-shifted from the 908 $cm^{-1}$ mode in the 555 nm Raman pump spectrum (**Figure 2b**). This observation is likely due to the enhancement of a shoulder peak at ~956 $cm^{-1}$ not resolved in the Stokes Raman spectrum, but apparent in the anti-Stokes FSRS spectrum (**Figure S5**) and assigned to similar bridge HOOP motions (**Table S4**) that could aid the photoinduced charge transfer events (Tang et al., 2018a).

For the excited-state ~1369 $cm^{-1}$ mode intensity decay dynamics of Cl-GFP and Br-GFP, both exhibit the similar hundreds of ps processes (**Figure 4c**). These characteristic time constants are shorter than the nanosecond (ns) time constants in fs-TA (**Table S6**) because of the reduced time window in FSRS (i.e., 250 ps < 600 ps in this work), while the vibrational intensity decay in FSRS is dependent on dynamic resonance enhancement as well (Oscar et al., 2017). A ~200 fs component is also retrieved from the least-squares fit, mainly due to the initial wavepacket motions along the photoinduced reaction coordinate near the Franck-Condon region of the Thr-Tyr-Gly (TYG) chromophore (Tang et al., 2015). This initial Franck-Condon relaxation is supported by the small but distinct rise component of the TA signal before 1 ps (see **Figure 4d** and **Table S6**) that usually leads to the stimulated emission (SE) peak redshift on ultrafast timescales immediately following actinic photoexcitation (Fang et al., 2019).

Notably, besides the excited-state FSRS peak dynamics plotted in **Figure 4c**, similar Raman intensity decay dynamics were also obtained for the decay of the Cl-GFP mode at 923 $cm^{-1}$ mode and the Br-GFP mode at 898 $cm^{-1}$ (**Figure S6**), which have strong carbon-halogen stretching contributions and are blue-shifted from their ground-state peak positions at 908 and 890 $cm^{-1}$, respectively (**Figure 2**, **Tables S4 and S5**). These modes exhibit a ~30 $cm^{-1}$ frequency blueshift (**Figure S6**) with similar single-exponential time constants as those retrieved from the Raman marker band intensity dynamics (**Figure 4c**), indicative of a vibrational cooling process of the conjugated chromophore that was previously observed in GFP-derived calcium biosensors (Tang et al., 2017; Tachibana et al., 2018). The halogen substituent may affect vibrational relaxation by modifying the electrostatic and steric interactions between the chromophore and surrounding internal water molecules and protein residues. A significant difference (~2.5 fold: 4.3 ps *versus* 10.8 ps) between the Cl-GFP and Br-GFP time constants, while both being much longer than the initial time constant (1.2 ps) of the unsubstituted sfGFP (**Table S6**), suggests that certain hindrance of vibrational cooling in Br-GFP leads to a decreased fluorescent population, hence a reduced fluorescence quantum yield (FQY) when compared with Cl-GFP (**Table S2**). In addition, the reduced long time constant of 0.98 ns in Br-GFP (*versus* 1.1 ns in Cl-GFP) is also consistent with a mixing with some nonradiative pathways and a smaller FQY of 0.76 (*versus* 0.88 in Cl-GFP) (Krueger et al., 2020a).

**Additional discussions of vibronic coupling of halogenated sfGFP derivatives.** In the steady-state emission spectra (**Figure 1**), the proteins exhibit a defined shoulder in the region probed by stimulated emission, 550–570 nm, which represents an energy difference of ~1400 $cm^{-1}$ to the fluorescence peak at ~515 nm (**Table S2**) (Tachibana et al., 2018). A shoulder also appears in the absorption spectra with a similar energy difference to the main absorption peak, suggesting that the origin of the feature is vibronic progression. Rigid aromatic systems and the π-stacked YFP typically display this behavior

(Wachter et al., 1998; Stavrov et al., 2006). The addition of a bulky halogen atom to the chromophore is expected to restrict chromophore motions to some extent, which may cause the vibronic feature to be more pronounced while the associated bridge and phenolate ring H-rocking motions become strongly coupled to the electronic transitions between $S_0$ and $S_1$. However, the relatively broad profile of the electronic shoulder peak indicates that the chromophore is not completely confined and exhibits the conformational heterogeneity. Overall, tighter packing inside the protein pocket may directly contribute to more efficient fluorescence as the deprotonated chromophore undergoes vibrational relaxation before radiative emission; we measured the FQYs of both Cl-GFP (0.88) and Br-GFP (0.76) to be higher than sfGFP (0.68) (Pédelacq et al., 2006) (see **Table S2**).

**Preliminary results of the transient absorption (TA) of nitro-GFP.** In difference from the halogenated sfGFP derivatives, nitro-GFP exhibits distinct TA dynamics that are associated with its minuscule fluorescence (**Table S2**). The broad and weak SE band is red-shifted (*versus* other proteins) and characterized by a rapid intensity loss. For the dynamics obtained on the red edge between 600–620 nm via the least-squares fit, the first decay time constant of ~3 ps (33% amplitude weight) is similar to other proteins (see **Figure 4d** in main text), but the second decay time constant of ~30 ps (67% weight) is indicative of nonradiative deactivation of the first singlet excited state ($S_1$). This process likely involves an efficient internal conversion back to the electronic ground state ($S_0$) via a conical intersection, facilitated by facile nuclear motions that involve the nitro substituent at the *ortho* site to the phenolate oxygen of the chromophore (Tang and Fang, 2019). Related work is currently ongoing in the laboratory and will be reported in a future publication.

# 2 Supplementary Tables and Figures

## 2.1 Supplementary Tables

**Supplementary Table 1.** Spectroscopic characterization of fluorescent protein (FP) chromophores using naturally occurring amino acids (top) and noncanonical amino acids (ncAAs, bottom)

**Natural chromophore modifications**

| Protein | Chromophore [a] | Absorption maximum (nm)[b] | Emission maximum (nm) | Fluorescence quantum yield (FQY)[c] | References |
|---|---|---|---|---|---|
| wtGFP | $S_{65}Y_{66}G_{67}$ | 395 (475) | 509 | 0.79 | (Brejc et al., 1997; Patterson et al., 1997; Fang et al., 2009) |
| EBFP2 | $S_{65}H_{66}G_{67}$ + Y145H | 383 | 448 | 0.56 | (Ai et al., 2007) |
| ECFP | $A_{65}W_{66}G_{67}$ or $T_{65}W_{66}G_{67}$ | 434 (452) | 477 (505) | 0.40 | (Heim and Tsien, 1996; Patterson et al., 2001) |
| EGFP | $T_{65}Y_{66}G_{67}$ | 488 (398) | 510 | 0.60 | (Patterson et al., 2001; Pal et al., 2005) |
| EYFP | $G_{65}Y_{66}G_{67}$ + $Y_{203}$ | 514 | 527 | 0.61 | (Patterson et al., 2001) |
| mCherry | $M_{65}Y_{66}G_{67}$ | 587 | 610 | 0.22 | (Shaner et al., 2004) |

**Noncanonical chromophore modifications**

| Y66 mutation of the protein chromophore[d] | Absorption maximum (nm)[b] | Emission maximum (nm) | References |
|---|---|---|---|
| *p*-amino-L-phenylalanine (SYG) | 435 | 498 | (Wang et al., 2003) |
| *p*-azido-L-phenylalanine (TYG) | 446 | 500 | (Reddington et al., 2013) |

| | | | |
|---|---|---|---|
| 3-fluoro-L-tyrosine (TYG) | 484 | 514 | (Pal et al., 2005) |
| 4-amino-Trp66 (TWG) | 466 | 574 | (Hyun Bae et al., 2003) |
| 3-amino-L-tyrosine (TYG) | 525 | 610 | (Augustine et al., 2019) |

[a] The chromophore residues are numbered by the wtGFP structure (PDB 1EMB) (Brejc et al., 1997). Key residue changes are listed for the chromophore, while some concomitant changes of surrounding protein residues are not highlighted here (Tsien, 1998; Patterson et al., 2001).

[b] Minor absorption bands are listed in parentheses, which typically correspond to a different protonation state of the chromophore.

[c] Quantum yield following excitation of the major absorption band.

[d] The chromophore composition is listed. Second row: TYG in sfGFP. Third and fifth rows: *av*GFP (enhanced green mutant or EGFP). Fourth row: GdFP ("gold" fluorescent protein).

**Supplementary Table 2.** Spectral parameters of fluorescent proteins (FPs) with site-specific ncAA incorporation at residue position 66

| Protein | Absorption maximum (nm)[a] | Emission maximum (nm)[b] | Stokes shift ($cm^{-1}$) | ε ($M^{-1}\cdot cm^{-1}$) | Fluorescence quantum yield[c] |
|---|---|---|---|---|---|
| sfGFP[d] | 395<br>488 | 508 (456)<br>510 | 5631 (3387)<br>884[e] | 20200<br>26600 | N/A<br>0.68 |
| Cl-GFP | 493 | 515 | 866[e] | 25800 | 0.88 |
| Br-GFP | 498 | 518 | 775[e] | 31300 | 0.76 |
| nitro-GFP | 415<br>478 | 558<br>520[f] (550) | 6175<br>1690 (2739) | 12600<br>24300 | N/A<br><0.0005 |

[a] When multiple absorption bands are present for the protein samples in pH=5.5 aqueous solution, the spectral features are ordered energetically from the highest to lowest. These absorption measurements are robust: we measured the absorption maxima of the nitro-GFP sample after more than five years of storage in a –20°C lab freezer, and obtained two absorption peak maxima at ca. 415 and 480 nm.

[b] Emission maxima were recorded using the protein sample absorption maximum as the excitation wavelength (see the vertical gray dotted lines in **Figure 1**). Shoulder peaks are listed in parentheses.

[c] Emission/fluorescence quantum yield (FQY) was measured using the relative method (Rurack and Spieles, 2011; Krueger et al., 2020a) following excitation of the major absorption band, and the fluorescein dye molecule (absorption/emission maximum at ~494/512 nm in water) with a wavelength-independent FQY of 0.93 was used as the standard (Sjöback et al., 1995). The average values of three independent measurements for individual samples are listed for a direct comparison between the protein samples, and one standard deviation is ca. ±0.03. Therefore, the experimentally obtained FQY increase from sfGFP to Br-GFP and then Cl-GFP is statistically significant.

[d] The residue numbering (i.e., Tyr66) in this column follows the superfolder GFP parent protein (Pédelacq et al., 2006).

[e] The relatively small Stokes shift is due to the excitation and detected emission of the deprotonated chromophore only (i.e., without involving the excited-state proton transfer for the protonated chromophores, see the other rows) (Chattoraj et al., 1996; Krueger et al., 2020b). The halogenation at the *ortho* site of the phenolate oxygen slightly decreases the Stokes shift in Cl-/Br-GFP *versus* sfGFP, likely due to the electron-withdrawing property, heavy mass, and steric hindrance of the chlorine or bromine substituent in comparison with the hydrogen atom.

[f] This bluer emission peak could be contaminated by the light scattering from buffer solution, mainly water (Chen et al., 2020) when the solute emission photon counts are very low. The main contribution from the nitro-GFP emission should therefore be above 550 nm.

**Supplementary Table 3.** Ground-state Raman mode assignment of the deprotonated chromophore of sfGFP based on DFT calculations

| Mode Assignment (major) | sfGFP experimental ($cm^{-1}$)[a] | Calculated freq. *in vacuo* ($cm^{-1}$)[b] | Calculated freq. in water ($cm^{-1}$)[b] |
|---|---|---|---|
| Phenolate ring in-plane deformation | 618 | 616 | 620 |
| Imid. ring in-plane deformation with small-scale phenolate ring deformation | 693 | 712 | 708 |
| Asymmetric phenolate ring H-out-of-plane (HOOP) wagging with ring deformation | 769 | 778 | 787 |
| Phenolate ring breathing with small-scale imid. ring in-plane deformation | 817 | 818 | 823 |
| Phenolate ring symmetric HOOP | 847 | 841 | 837 |
| Phenolate ring carbon OOP with phenolate ring and bridge HOOP | 914 | 927 | 928 |
| Phenolate ring H- and bridge H-scissoring | 1083 | 1081 | 1086 |
| Phenolate ring H-scissoring, bridge H-rocking, and imid. ring in-plane deformation | 1167 | 1157 | 1153 |
| Phenolate ring H-rock and CO stretch, and bridge CCC bend with H-rocking | 1256 | 1252 | 1250 |
| Bridge and phenyl ring H-rocking, and phenolate ring in-plane deformation | 1330 | 1320 | 1308 |
| Imid. ring C–N stretch with phenolate ring and bridge H-rocking | 1369 | 1367 | 1365 |
| Phenolate CC stretch, phenyl ring H-rocking | 1452 | 1453 | 1452 |
| Asymmetric phenolate C=C stretch and phenolate C=O stretch | 1500 | 1495 | 1480 |
| Imid. ring C=N stretch, bridge C=C stretch, and phenolate C=O stretch | 1547 | 1545 | 1543 |
| Phenolate ring symmetric C=C stretch and C=O stretch, and bridge C=C stretch | 1621 | 1623 | 1614 |

[a] The Raman peak frequencies obtained from the ground-state FSRS measurement (see **Figure 2a**).

[b] The calculated Raman mode frequencies are with a scaling factor of 0.98 after ground-state density functional theory (DFT) calculations at the RB3LYP level with 6-311G+(d, p) basis sets. Such DFT calculations are typical and reliable for the GFP chromophores, including the three-residue SYG and TYG configurations (Schellenberg et al., 2001; Tang et al., 2016; Taylor et al., 2019). A detailed comparison between the observed and calculated Raman modes can then aid the mode assignment and experimental spectral interpretation (see main text). Note for the deprotonated TYG chromophore, the phenyl or phenolate ring refers to the same structural moiety while the latter highlights the $–O^{-}$ group.

**Supplementary Table 4.** Ground-state Raman mode assignment of the deprotonated chromophore of Cl-GFP based on DFT calculations

| Mode Assignment (major) | Cl-GFP experimental ($cm^{-1}$)[a] | Calculated freq. *in vacuo* ($cm^{-1}$)[b] | Calculated freq. in water ($cm^{-1}$)[b] |
|---|---|---|---|
| Phenolate ring translation and CCO bending with C–Cl stretch | 541 | 535[c] | 535[c] |
| | | 536[d] | 536[d] |
| Phenolate ring in-plane deformation with C–Cl stretch | 623 | 641 | 646 |
| | | 647 | 653 |
| Imid. ring OOP deformation | 699[e] | 706 | 701 |
| | | 705 | 699 |
| Imid. ring in-plane deformation with some phenolate ring deformation | 766 | 761 | 757 |
| | | 765 | 760 |
| Phenolate ring breathing with C–Cl stretch[f] | 908 | 866 | 869 |
| | | 882 | 884 |
| Phenolate ring and bridge symmetric HOOP | 956[e] | 944 | 939 |
| | | 955 | 959 |
| Phenolate ring H-scissoring with bridge H-rocking | 1079 | 1103 | 1100 |
| | | 1107 | 1115 |
| Phenolate ring H-scissoring with CCO rocking motion | 1191 | 1196 | 1201 |
| | | 1196 | 1198 |
| Phenolate ring H-rock, bridge CCC bend with H-rock, and imid. ring in-plane deformation | 1261 | 1260 | 1252 |
| | | 1263 | 1257 |
| Imid. ring asymmetric deformation and phenolate ring H-rocking | 1299 | 1291 | 1286 |
| | | 1291 | 1289 |
| Imid. ring C–N stretch with phenolate ring and bridge H-rocking | 1360 | 1352 | 1355 |
| | | 1367 | 1361 |
| Asymmetric phenyl C=C stretch and phenolate C=O stretch | 1486 | 1502 | 1499 |
| | | 1500 | 1485 |

| | | | |
|---|---|---|---|
| Imid. ring C=N stretch, bridge C=C stretch, and phenolate C=O stretch | 1542 | 1543 | 1541 |
| | | 1540 | 1542 |
| Phenolate C=O and C=C stretch, bridge C=C stretch, and imid. ring C=N stretch | 1577[e] | 1571 | 1575 |
| | | 1569 | 1573 |
| Phenolate ring symmetric C=C stretch, phenolate C=O stretch, and bridge C=C stretch | 1620 | 1623 | 1615 |
| | | 1624 | 1615 |

[a] The Raman peak frequencies obtained from the ground-state FSRS measurement (see **Figure 2b**).

[b] The calculated Raman mode frequencies with a scaling factor of 0.98 after ground-state DFT calculations at the RB3LYP level with 6-311G+(d, p) basis sets.

[c] The top row (in this entire column) is the mode frequency calculated for Configuration 1 (see **Figure 3**, X=Cl).

[d] The bottom row (in this entire column) is the mode frequency calculated for Configuration 2 (see **Figure 3**, X=Cl).

[e] These relatively weak shoulder peaks in **Figure 2b** are better resolved in the pre-resonance Stokes FSRS spectrum with a bluer 507 nm Raman pump (**Figure S4**, red trace) as well as the anti-Stokes FSRS spectrum with a redder 580 nm Raman pump (**Figure S5**, red trace).

[f] The magnitude of this mode frequency blueshift from the unsubstituted sfGFP chromophore (see **Table S3**) is dependent on the chromophore conformation, in particular, the position of the chlorine substituent. However, due to the broadness of Raman peaks in **Figure 2b**, normal modes from various conformations could have overlapping vibrational features so the predicted mode frequency shift by itself cannot lead to a definitive assignment of one chromophore conformational state over the other (see **Figure 3**, X=Cl).

**Supplementary Table 5.** Ground-state Raman mode assignment of the deprotonated chromophore of Br-GFP based on DFT calculations

| Mode Assignment | Br-GFP experimental ($cm^{-1}$)[a] | Calculated freq. *in vacuo* ($cm^{-1}$)[b] | More calculated freq. *in vacuo* ($cm^{-1}$)[c] | Calculated freq. in water ($cm^{-1}$)[b] |
|---|---|---|---|---|
| Phenolate ring translation and CCO bending with some C–Br stretch | 530 | 528[d] | 530 | 526 |
| | | 518[e] | | 517 |
| Phenolate ring in-plane deformation with some C–Br stretch | 623 | 629 | 632 | 633 |
| | | 633 | | 638 |
| Imid. ring OOP deformation | 699 | 705 | 706 | 701 |
| | | 702 | | 699 |
| Imid. ring in-plane deformation with some phenyl ring deformation | 768 | 761 | 763 | 757 |
| | | 754 | | 748 |
| Phenolate ring one-side symmetric HOOP | 820 | 819 | 821 | 815 |
| | | 804 | | 806 |
| Phenolate ring breathing with C–Br stretch[f] | 890 | 852 | 855 | 860 |
| | | 861 | | 864 |
| Phenolate ring carbon OOP with phenolate ring and bridge HOOP | 918 | 881 | 887 | 883 |
| | | 920 | | 925 |
| Phenolate ring H-scissoring with bridge H-rocking | 1084 | 1100 | 1102 | 1100 |
| | | 1097 | | 1106 |
| Phenolate ring H-scissoring with CCO in-plane bending | 1192 | 1197 | 1201 | 1203 |
| | | 1195 | | 1196 |
| Phenolate ring H-rock, bridge CCC bend with H-rock, and imid. ring in-plane deformation | 1261 | 1260 | 1264 | 1254 |
| | | 1265 | | 1261 |
| Phenolate ring in-plane deformation and ring H-rocking | 1298 | 1315 | 1319 | 1305 |
| | | 1286 | | 1288 |
| Imid. ring C–N stretch with phenolate ring and bridge H-rocking | 1361 | 1350 | 1353 | 1353 |
| | | 1359 | | 1360 |

| | | | | |
|---|---|---|---|---|
| Asymmetric phenyl C=C stretch and phenolate C=O stretch | 1483 | 1495 | 1498 | 1480 |
| | | 1492 | | 1477 |
| Imid. ring C=N stretch, bridge C=C stretch, and phenolate C=O stretch | 1541 | 1543 | 1544 | 1541 |
| | | 1541 | | 1542 |
| Phenolate C=O and C=C stretch, and imid. ring C=N stretch | 1576[g] | 1572 | 1575 | 1575 |
| | | 1571 | | 1574 |
| Phenolate ring symmetric C=C stretch, phenolate C=O stretch, and bridge C=C stretch | 1622 | 1620 | 1621 | 1612 |
| | | 1624 | | 1612 |

[a] The Raman peak frequencies obtained from the ground-state FSRS measurement (see **Figure 2c**).

[b] The calculated Raman mode frequencies with a scaling factor of 0.98 after ground-state DFT calculations at the RB3LYP level with 6-311G+(d, p) basis sets.

[c] In this extra middle column, the RB3LYP calculations were performed with expanded basis sets, 6-311G++(df, pd), on Configuration 1 to account for the Br orbitals. However, the calculated frequencies were not found to be significantly different from those predicted by the lower level of theory (listed in the column to the left). Hence the same calculations were not performed for Configuration 2 in this middle column.

[d] The top row (in this entire column) is the mode frequency calculated for Configuration 1 (see **Figure 3**, X=Br).

[e] The bottom row (in this entire column) is the mode frequency calculated for Configuration 2 (see **Figure 3**, X=Br).

[f] The magnitude of this mode frequency blueshift from the unsubstituted sfGFP chromophore (see **Table S3**) is dependent on the chromophore conformation, in particular, the position of the bromine substituent. However, due to the broadness of Raman peaks in **Figure 2c**, normal modes from various conformations could have overlapping vibrational features so the predicted mode frequency shift by itself cannot lead to a definitive assignment of one chromophore conformational state over the other (see **Figure 3**, X=Br). In this case, the adjacent 918 $cm^{-1}$ mode also involves Br motions on the phenolate ring, and therefore the energy gap between the calculated modes is a more reliable measure.

[g] This relatively weak shoulder peak in **Figure 2c** is better resolved in the pre-resonance Stokes FSRS spectrum with a bluer 507 nm Raman pump (**Figure S4**, blue trace) as well as the anti-Stokes FSRS spectrum with a redder 580 nm Raman pump (**Figure S5**, blue trace).

**Supplementary Table 6.** Least-squares fit parameters for the stimulated emission band decay dynamics from 550−570 nm following 480 nm excitation[a]

| Protein | $A_1$ (%) | $\tau_1$ (ps) | $A_2$ (%) | $\tau_2$ (ns) |
|---|---|---|---|---|
| sfGFP | 20 | 1.2 | 80 | 1.2 |
| Cl-GFP | 37 | 4.1 | 63 | 1.1 |
| Br-GFP | 27 | 12.4 | 73 | 0.98 |

[a] For each protein sample, the stimulated emission (SE) band integrated signal from 550–570 nm is fit with an additional ~150 fs time component to account for the initial signal reaching the maximal SE intensity magnitude (i.e., a negative signal in a transient absorption experiment) before the ensuing decay on the picosecond (ps) to nanosecond (ns) timescales toward zero OD. The initial rise component is responsible for the SE band peaking at ~600 fs, likely due to the Franck-Condon dynamics that rapidly populate the initial fluorescent state of the deprotonated TYG chromophore inside sfGFP (Tang et al., 2018b). The initial decay of the SE signal, on the other hand, probes the vibrational cooling process as the chromophore navigates the excited state potential energy surface toward the lower-lying fluorescent state (see main text). Notably, the oscillator strength of the SE downward transition could change as energy relaxation occurs for a photoexcited protein chromophore (Kumpulainen et al., 2017; Fang and Tang, 2020). Though the majority of the SE decay involves the fluorescence pathway on the ns timescale that depletes the excited-state population, the ultrafast SE decay on the ps timescale implies that the maximal SE oscillator strength occurs in close proximity to the Franck-Condon region upon electronic excitation of the protein TYG chromophore (see **Figure 4d** inset).

## 2.2 Supplementary Figures

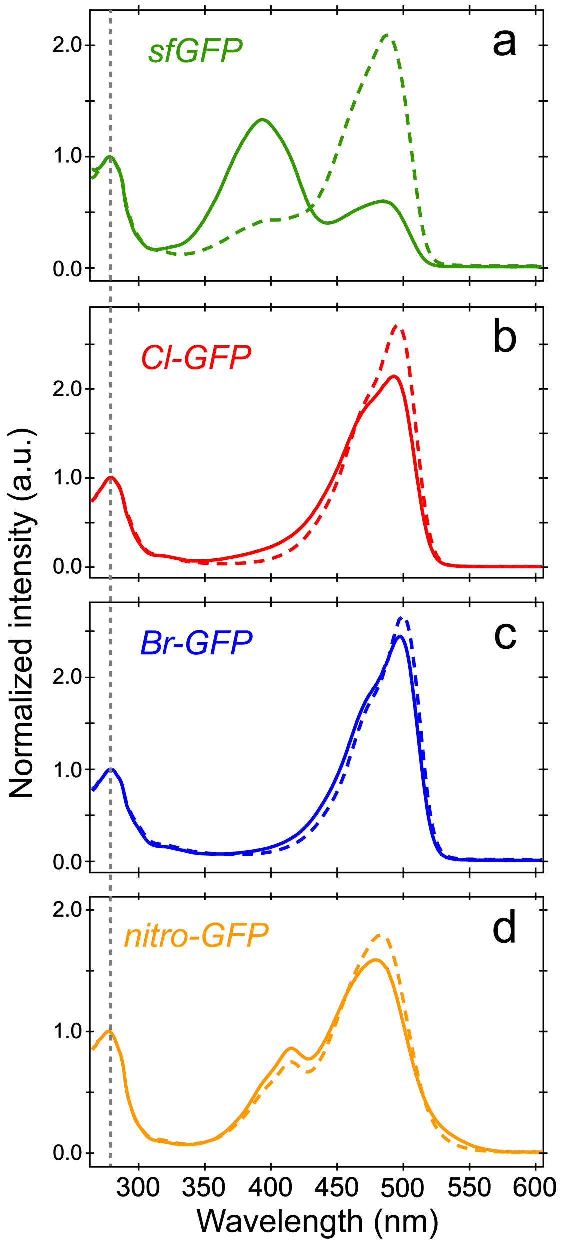


**Supplementary Figure 1.** The steady-state electronic absorption spectra of (a) sfGFP, (b) Cl-GFP, (c) Br-GFP, and (d) nitro-GFP in pH=5.5 (solid lines) and pH=8.1 (dashed lines) buffer solutions. The spectra are normalized at 279 nm (vertical dotted line) for comparison. At lower pH, the proteins exhibit higher absorption intensities below 450 nm where the neutral chromophore absorbs. The most significant pH-dependent spectral difference occurs in sfGFP (i.e., with a significant deprotonation of the TYG chromophore) (Pédelacq et al., 2006), while the deprotonation chromophore population increases to various extent in all four samples (all with the TYG chromophore).

Notably, the halogenated sfGFP shows a much reduced neutral chromophore population at pH=5.5, and the associated absorption peak around 400 nm is further diminished at pH=8.1 (see the solid to dashed trace in panels **b** and **c**). These results indicate a much lower p$K_a$ (<5) of the TYG chromophore phenolic hydroxyl inside the halogenated sfGFP than that inside wild-type sfGFP (Shu et al., 2007).

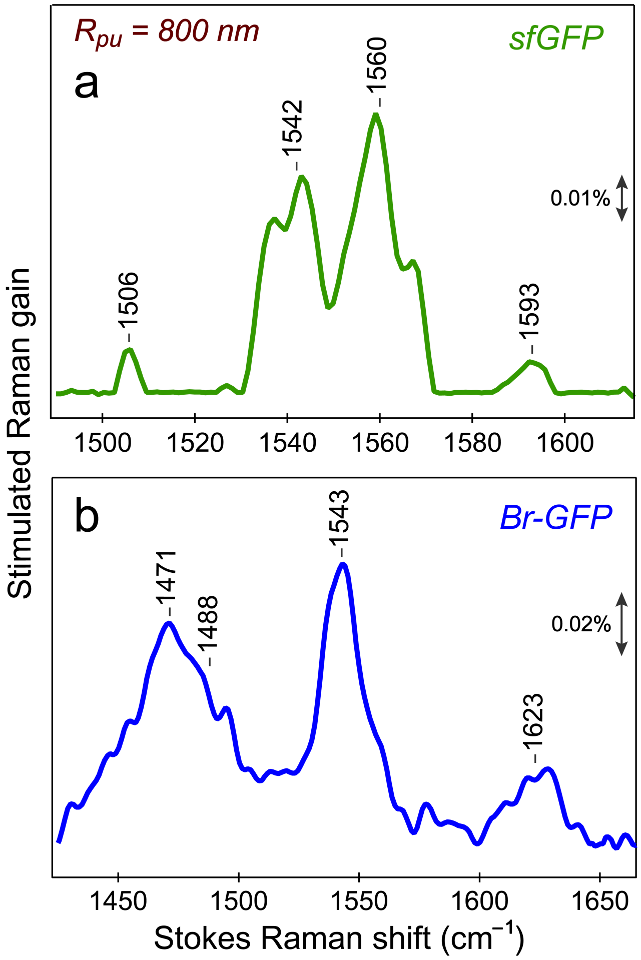


**Supplementary Figure 2.** Ground-state FSRS on the Stokes side with an 800 nm Raman pump and white light probe for (a) sfGFP and (b) Br-GFP in pH=5.5 aqueous buffer solution. The double-headed arrow indicates the magnitude of the stimulated Raman gain. Vibrational marker bands for both the neutral chromophore (1560 cm$^{-1}$) and anionic chromophore (1542 cm$^{-1}$) are observed in sfGFP (Bell et al., 2000; Taylor et al., 2019), whereas only the anionic chromophore marker band (1543 cm$^{-1}$) is present in Br-GFP, indicative of its lower p$K_a$ than that inside sfGFP (see main text). These vibrational signatures are in accord with the observed steady-state electronic spectra in **Figure S1a** and **c**, respectively.

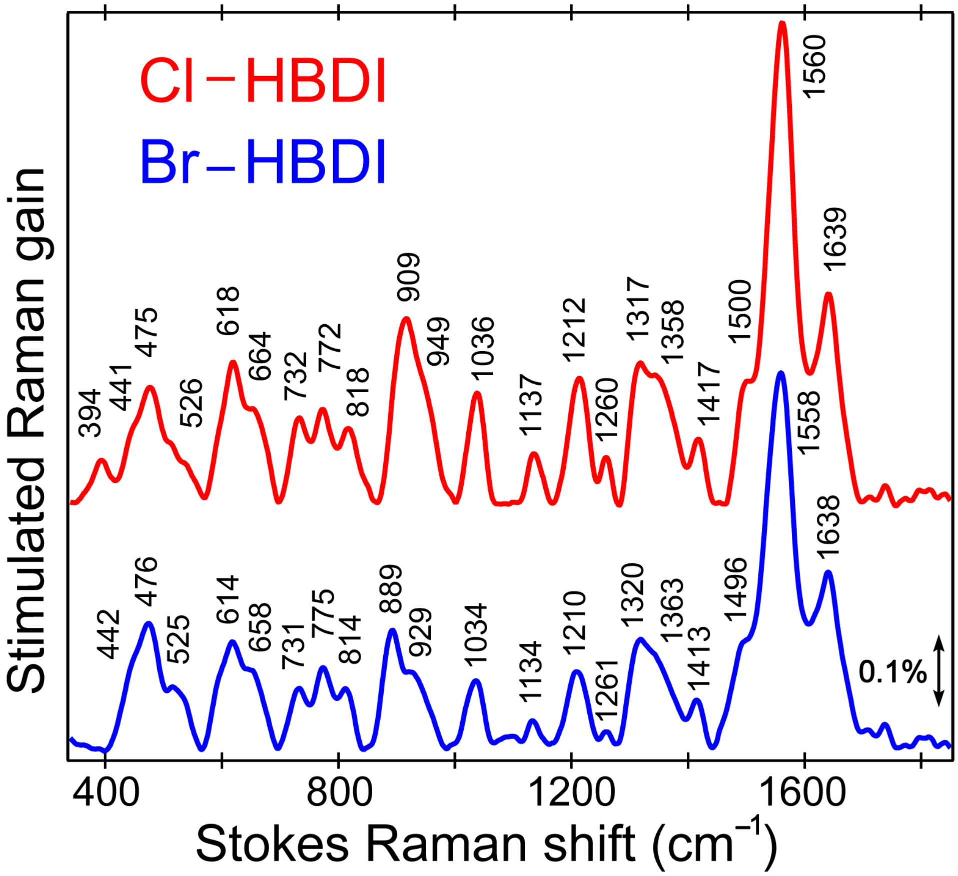


**Supplementary Figure 3.** Ground-state Stokes FSRS data of the Cl-HBDI (red) and Br-HBDI (blue) anionic model chromophores (in pH=7.6 aqueous solution) with a 480 nm Raman pump and white light probe. The double-headed arrow indicates the stimulated Raman gain magnitude of 0.1%.

Given the main electronic absorption bands at ~425 nm for Cl- and Br-HBDI, the pre-resonance enhancement condition was achieved by the 480 nm Raman pump to improve the signal-to-noise ratio in these experiments (Chen et al., 2018; Fang et al., 2019). Notable differences exist between these vibrational peaks of the anionic form halogenated HBDIs and the unsubstituted HBDI (Bell et al., 2000; Taylor et al., 2019) in aqueous solution. As the Raman pump wavelength is further tuned away from the absorption peak of the chromophore (e.g., toward the redder side), the Raman peak width typically decreases (Chen et al., 2018; Fang et al., 2019) so the overlapping bands in the pre-resonance spectra (see **Figure S3** above) can become better resolved. However, the pre-resonance condition here enables the vibrational modes with strong vibronic coupling to be enhanced more than other modes (Myers and Mathies, 1987; Quick et al., 2015; Fang et al., 2018), which is beneficial to identify key modes in the Franck-Condon region.

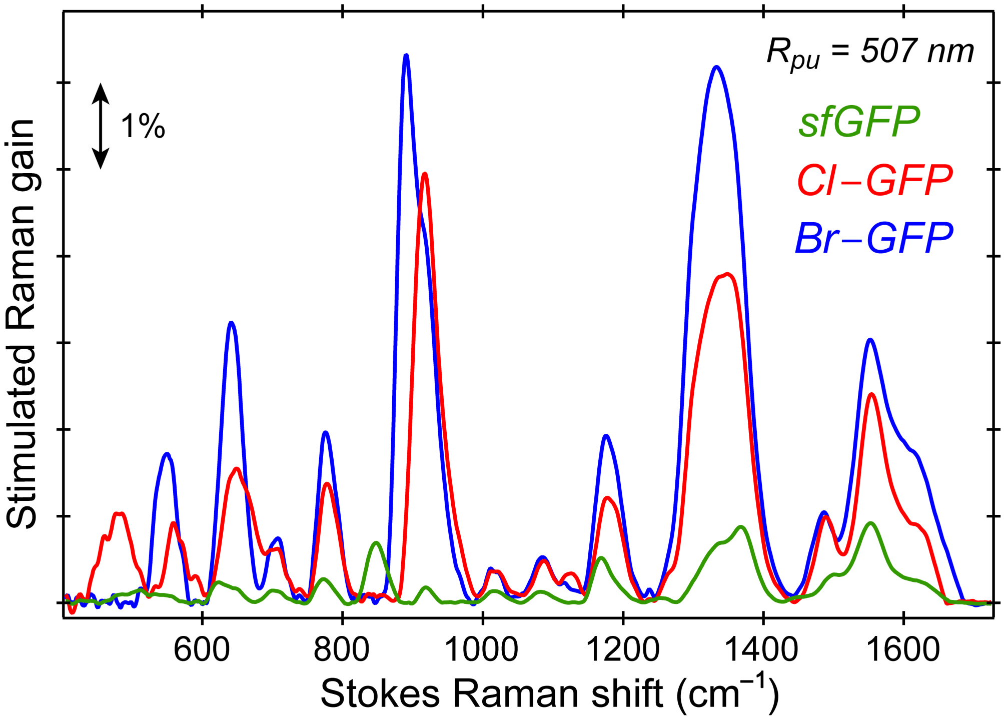


**Supplementary Figure 4.** Pre-resonance FSRS spectrum of 10 mg/mL sfGFP (green), Cl-GFP (red), and Br-GFP (blue) in pH=5.5 aqueous buffer solution using a 507 nm Raman pump. The magnitude of the stimulated Raman gain at 1% is depicted by the double-headed arrow. In reference to sfGFP, the low-frequency modes of Cl-GFP and Br-GFP are enhanced by a larger degree relative to the high-frequency modes (>1000 cm$^{-1}$).

The Cl- and Br-GFP absorption bands overlap more with the Raman pump (see **Figures 1** and **S1**), which contributes to the much increased signal strength of the halogenated GFP samples. The Cl-GFP spectrum shows a new peak at ~480 cm$^{-1}$, while the peak at ~704 cm$^{-1}$ is better resolved compared to the 555 nm Raman pump ground state spectrum (see **Figure 2b**) due to the change of resonance conditions (Fang et al., 2018; Fang and Tang, 2020). The broadness of vibrational peaks is due to the Raman pump being very close to the ground-state absorption band ($S_0 \rightarrow S_1$) so the directly stimulated Raman peaks in the electronic excited state (i.e., within the Franck-Condon region) become possible (Quick et al., 2015; Chen et al., 2018). The slight mismatch between peak frequencies here and those in **Figure 2** (i.e., the redder Raman pump is further away from the electronic absorption band by ~50 nm) could therefore be due to a mixture of $S_0$ and $S_1$ Raman bands. A major contributor to the observed mode frequency shift is thus attributed to initial electronic redistribution upon photoexcitation (by the picosecond Raman pump pulse in this case) (Fang et al., 2009; Oscar et al., 2014).

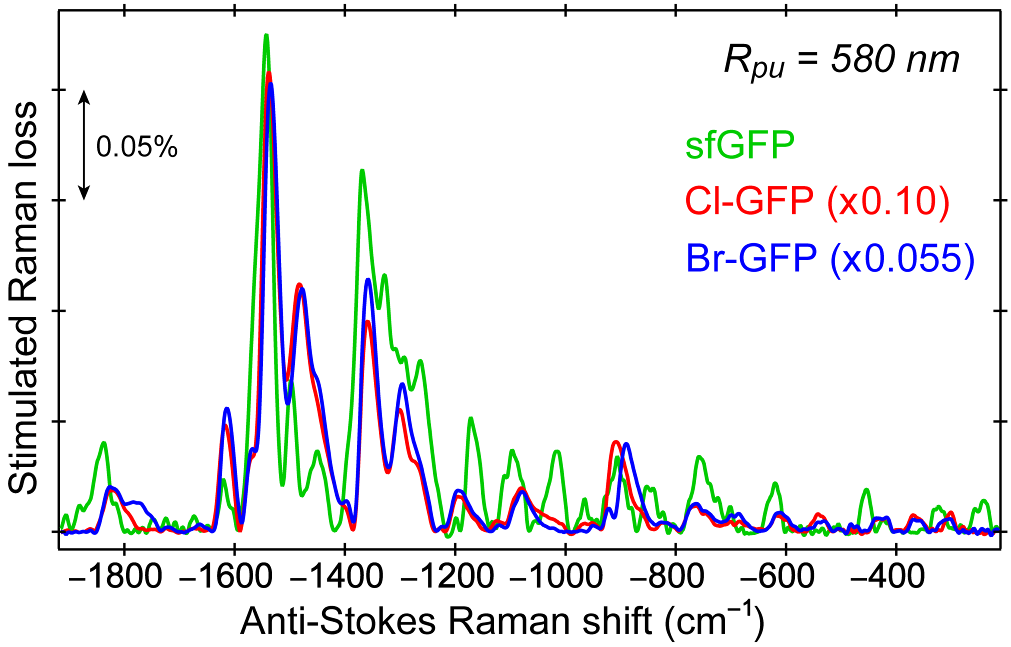


**Supplementary Figure 5.** Ground-state anti-Stokes FSRS spectra of sfGFP (green), Cl-GFP (red), and Br-GFP (blue) in pH=5.5 aqueous buffer solution. The Raman pump was tuned to 580 nm. The intensity scaling factors are noted in the parentheses to enable a direct comparison between the much stronger Raman peaks for Cl-/Br-GFP and the weak Raman peaks of sfGFP.

The significantly enhanced Raman peaks of Cl-GFP in comparison to sfGFP is due to a dominant deprotonated chromophore population in Cl-GFP and its red-shifted major absorption peak at 493 nm, which is closer to the 580 nm Raman pump than the 488 nm minor absorption peak in sfGFP (see **Figure 1**). The further enhanced Raman peaks of Br-GFP can be attributed to its further red-shifted major absorption peak at 498 nm that gets even closer to the 580 nm Raman pump, and an increased electric polarizability due to the larger size of Br (Liu et al., 2016; Chen et al., 2020). In addition, owing to a better overlap between the bluer Raman probe and the electronic absorption band at the ground state (e.g., with 580 nm Raman pump the ~1543 cm$^{-1}$ mode of sfGFP corresponds to the Raman probe wavelength at ~532 nm, which approaches the red edge of the ground state absorption band), the high-frequency modes become more enhanced than the low-frequency modes in the anti-Stokes spectrum (Chen et al., 2018; Tang et al., 2018a).

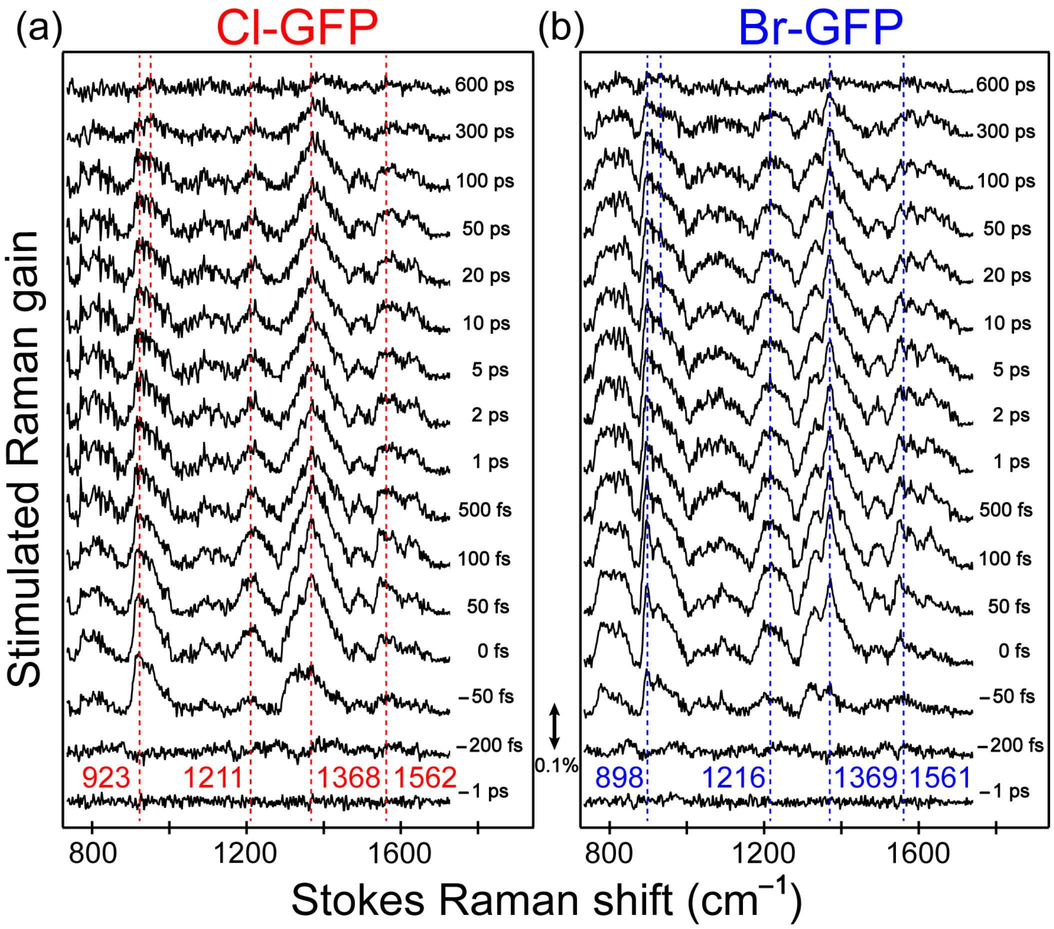


**Supplementary Figure 6.** Time-resolved excited-state FSRS data of (a) Cl-GFP and (b) Br-GFP in pH=5.5 aqueous buffer solution with a 555 nm Raman pump following photoexcitation at 480 nm. The stimulated Raman gain of 0.1% is indicated by the double-headed arrow between two panels. Time delay points are noted to the right side of each spectrum from –1 to 600 ps. The vertical dashed lines in red and blue highlight the initial (i.e., around the time zero of photoexcitation) Raman marker band frequencies in panels (a) and (b), respectively, wherein a small but noticeable frequency blueshift occurs at later time points likely due to vibrational cooling within the $S_1$ state of the deprotonated chromophore (see **Figure S1** for the relative position of the anionic chromophore absorption with respect to the 480 nm actinic pump) (Tang et al., 2017; Fang and Tang, 2020). These blue-shifted Raman peaks are highlighted by shorter vertical dashed lines at later time points (>5 ps) in both panels.

The specific Raman pump wavelength achieves a pre-resonance condition with the red edge of the stimulated emission band (Tang et al., 2016; Tang et al., 2018a) to enhance the excited-state vibrational features as shown for the deprotonated chromophore. The notable mode frequency blueshift from $S_0$ to $S_1$ (T=0 fs), e.g., ~1360 to 1368 cm$^{-1}$, and 1542 to 1562 cm$^{-1}$ (see **Figures 2b-c** and **4a-b**), indicates that a photoinduced electron redistribution occurs over the chromophore ring system and certain modes exhibit more polarizability change than others. Based on the mode assignment from DFT calculations of a deprotonated TYG chromophore (see **Tables S3-S5** above), the 1360 cm$^{-1}$ mode corresponds to the imidazolinone C–N stretching and phenolate ring/bridge H-rocking motions, while the 1542 cm$^{-1}$ mode is associated with the imidazolinone C=N stretch, bridge C–C=C stretch, and phenolate C=O stretching motions (Tang et al., 2016). The incorporation of an electron-withdrawing halogen atom at

the *ortho* site to the phenolate –C=O$^{(-)}$ group thus exerts a discernible effect on the chromophore conjugated ring system, and the electrostatic effect could supersede the steric effect on the sub-picosecond timescale due to the intrinsic mass difference between electrons and nuclei. In particular, after actinic electronic excitation, those high-frequency stretching and rocking motions of the chromophore are strengthened to some extent and become active starting from the Franck-Condon region, as observed in **Figure 4a-b** and **Figure S6**. Moreover, the much broader linewidth of the $S_1$ Raman peaks than the $S_0$ peaks arises from the transient nature of the electronic excited state (i.e., shorter lifetime hence larger uncertainty in the energy measurement) and the potential conformational inhomogeneity of the photoexcited TYG chromophore (Tang et al., 2016; Fang and Tang, 2020).

## 3 Supplementary References

Ai, H.-w., Shaner, N. C., Cheng, Z., Tsien, R. Y., and Campbell, R. E. (2007). Exploration of new chromophore structures leads to the identification of improved blue fluorescent proteins. *Biochemistry* 46(20)**,** 5904-5910. doi: 10.1021/bi700199g.

Augustine, G., Raghavan, S., NumbiRamudu, K., Easwaramoorthi, S., Shanmugam, G., Seetharani Murugaiyan, J., et al. (2019). Excited state electronic interconversion and structural transformation of engineered red-emitting green fluorescent protein mutant. *J. Phys. Chem. B* 123(10)**,** 2316-2324. doi: 10.1021/acs.jpcb.8b10516.

Bell, A. F., He, X., Wachter, R. M., and Tonge, P. J. (2000). Probing the ground state structure of the green fluorescent protein chromophore using Raman spectroscopy. *Biochemistry* 39(15)**,** 4423-4431. doi: 10.1021/bi992675o.

Brejc, K., Sixma, T. K., Kitts, P. A., Kain, S. R., Tsien, R. Y., Ormö, M., et al. (1997). Structural basis for dual excitation and photoisomerization of the *Aequorea victoria* green fluorescent protein. *Proc. Natl. Acad. Sci. U.S.A.* 94(6)**,** 2306-2311. doi: 10.1073/pnas.94.6.2306.

Chattoraj, M., King, B. A., Bublitz, G. U., and Boxer, S. G. (1996). Ultra-fast excited state dynamics in green fluorescent protein: multiple states and proton transfer. *Proc. Natl. Acad. Sci. U.S.A.* 93(16)**,** 8362-8367. doi: 10.1073/pnas.93.16.8362.

Chen, C., Baranov, M. S., Zhu, L., Baleeva, N. S., Smirnov, A. Y., Zaitseva, S., et al. (2019). Designing redder and brighter fluorophores by synergistic tuning of ground and excited states. *Chem. Commun.* 55(17)**,** 2537-2540. doi: 10.1039/C8CC10007A.

Chen, C., Zhu, L., Boulanger, S. A., Baleeva, N. S., Myasnyanko, I. N., Baranov, M. S., et al. (2020). Ultrafast excited-state proton transfer dynamics in dihalogenated non-fluorescent and fluorescent GFP chromophores. *J. Chem. Phys.* 152(2)**,** 021101. doi: 10.1063/1.5138666.

Chen, C., Zhu, L., and Fang, C. (2018). Femtosecond stimulated Raman line shapes: dependence on resonance conditions of pump and probe pulses. *Chin. J. Chem. Phys.* 31(4)**,** 492-502. doi: 10.1063/1674-0068/31/cjcp1805125.

Fang, C., Frontiera, R. R., Tran, R., and Mathies, R. A. (2009). Mapping GFP structure evolution during proton transfer with femtosecond Raman spectroscopy. *Nature* 462(7270)**,** 200-204. doi: 10.1038/nature08527.

Fang, C., and Tang, L. (2020). Mapping structural dynamics of proteins with femtosecond stimulated Raman spectroscopy. *Annu. Rev. Phys. Chem.* 71(1). doi: 10.1146/annurev-physchem-071119-040154.

Fang, C., Tang, L., and Chen, C. (2019). Unveiling coupled electronic and vibrational motions of chromophores in condensed phases. *J. Chem. Phys.* 151(20)**,** 200901. doi: 10.1063/1.5128388.

Fang, C., Tang, L., Oscar, B. G., and Chen, C. (2018). Capturing structural snapshots during photochemical reactions with ultrafast Raman spectroscopy: from materials transformation to biosensor responses. *J. Phys. Chem. Lett.* 9(12)**,** 3253–3263. doi: 10.1021/acs.jpclett.8b00373.

Heim, R., and Tsien, R. Y. (1996). Engineering green fluorescent protein for improved brightness, longer wavelengths and fluorescence resonance energy transfer. *Curr. Biol.* 6(2)**,** 178-182. doi: 10.1016/S0960-9822(02)00450-5.

Hyun Bae, J., Rubini, M., Jung, G., Wiegand, G., Seifert, M. H. J., Azim, M. K., et al. (2003). Expansion of the genetic code enables design of a novel "gold" class of green fluorescent proteins. *J. Mol. Biol.* 328(5)**,** 1071-1081. doi: 10.1016/S0022-2836(03)00364-4.

Kojima, S., Ohkawa, H., Hirano, T., Maki, S., Niwa, H., Ohashi, M., et al. (1998). Fluorescent properties of model chromophores of tyrosine-66 substituted mutants of *Aequorea* green fluorescent protein (GFP). *Tetra. Lett.* 39(29)**,** 5239-5242. doi: 10.1016/S0040-4039(98)01031-4.

Krueger, T. D., Boulanger, S. A., Zhu, L., Tang, L., and Fang, C. (2020a). Discovering a rotational barrier within a charge-transfer state of a photoexcited chromophore in solution. *Struct. Dyn.* 7(2)**,** 024901. doi: 10.1063/1.5143441.

Krueger, T. D., Tang, L., Zhu, L., Breen, I. L., Wachter, R. M., and Fang, C. (2020b). Dual illumination enhances transformation of an engineered green-to-red photoconvertible fluorescent protein. *Angew. Chem. Int. Ed.* 59(4)**,** 1644-1652. doi: 10.1002/anie.201911379.

Kumpulainen, T., Lang, B., Rosspeintner, A., and Vauthey, E. (2017). Ultrafast elementary photochemical processes of organic molecules in liquid solution. *Chem. Rev.* 117(16)**,** 10826-10939. doi: 10.1021/acs.chemrev.6b00491.

Liu, W., Wang, Y., Tang, L., Oscar, B. G., Zhu, L., and Fang, C. (2016). Panoramic portrait of primary molecular events preceding excited state proton transfer in water. *Chem. Sci.* 7(8)**,** 5484-5494. doi: 10.1039/C6SC00672H.

Myers, A. B., and Mathies, R. A. (1987). "Resonance Raman Intensities: A Probe of Excited-State Structure and Dynamics," in *Biological Applications of Raman Spectroscopy,* ed. T.G. Spiro. (New York: John Wiley & Sons, Inc.), 1-58.

Oscar, B. G., Chen, C., Liu, W., Zhu, L., and Fang, C. (2017). Dynamic Raman line shapes on an evolving excited-state landscape: insights from tunable femtosecond stimulated Raman spectroscopy. *J. Phys. Chem. A* 121(29)**,** 5428-5441. doi: 10.1021/acs.jpca.7b04404.

Oscar, B. G., Liu, W., Zhao, Y., Tang, L., Wang, Y., Campbell, R. E., et al. (2014). Excited-state structural dynamics of a dual-emission calmodulin-green fluorescent protein sensor for calcium ion imaging. *Proc. Natl. Acad. Sci. U.S.A.* 111(28)**,** 10191-10196. doi: 10.1073/pnas.1403712111.

Pal, P. P., Bae, J. H., Azim, M. K., Hess, P., Friedrich, R., Huber, R., et al. (2005). Structural and spectral response of *Aequorea victoria* green fluorescent proteins to chromophore fluorination. *Biochemistry* 44(10)**,** 3663-3672. doi: 10.1021/bi0484825.

Patterson, G., Day, R. N., and Piston, D. (2001). Fluorescent protein spectra. *J. Cell Sci.* 114(5)**,** 837-838.

Patterson, G. H., Knobel, S. M., Sharif, W. D., Kain, S. R., and Piston, D. W. (1997). Use of the green fluorescent protein and its mutants in quantitative fluorescence microscopy. *Biophys. J.* 73(5)**,** 2782-2790. doi: 10.1016/S0006-3495(97)78307-3.

Pédelacq, J.-D., Cabantous, S., Tran, T., Terwilliger, T. C., and Waldo, G. S. (2006). Engineering and characterization of a superfolder green fluorescent protein. *Nat. Biotechnol.* 24(1)**,** 79-88. doi: 10.1038/nbt1172.

Quick, M., Dobryakov, A. L., Kovalenko, S. A., and Ernsting, N. P. (2015). Resonance femtosecond-stimulated Raman spectroscopy without actinic excitation showing low-frequency vibrational

activity in the S2 state of all-trans β-carotene. *J. Phys. Chem. Lett.* 6(7), 1216-1220. doi: 10.1021/acs.jpclett.5b00243.

Reddington, S. C., Rizkallah, P. J., Watson, P. D., Pearson, R., Tippmann, E. M., and Jones, D. D. (2013). Different photochemical events of a genetically encoded phenyl azide define and modulate GFP fluorescence. *Angew. Chem. Int. Ed.* 52(23), 5974-5977. doi: 10.1002/anie.201301490.

Rurack, K., and Spieles, M. (2011). Fluorescence quantum yields of a series of red and near-infrared dyes emitting at 600−1000 nm. *Anal. Chem.* 83(4), 1232-1242. doi: 10.1021/ac101329h.

Schellenberg, P., Johnson, E., Esposito, A. P., Reid, P. J., and Parson, W. W. (2001). Resonance Raman scattering by the green fluorescent protein and an analogue of its chromophore. *J. Phys. Chem. B* 105(22), 5316-5322. doi: 10.1021/jp0046243.

Shaner, N. C., Campbell, R. E., Steinbach, P. A., Giepmans, B. N. G., Palmer, A. E., and Tsien, R. Y. (2004). Improved monomeric red, orange and yellow fluorescent proteins derived from *Discosoma* sp. red fluorescent protein. *Nat. Biotechnol.* 22(12), 1567-1572. doi: 10.1038/nbt1037.

Shu, X., Kallio, K., Shi, X., Abbyad, P., Kanchanawong, P., Childs, W., et al. (2007). Ultrafast excited-state dynamics in the green fluorescent protein variant S65T/H148D. 1. mutagenesis and structural studies. *Biochemistry* 46(43), 12005-12013. doi: 10.1021/bi7009037.

Sjöback, R., Nygren, J., and Kubista, M. (1995). Absorption and fluorescence properties of fluorescein. *Spectrochim. Acta A: Mol. Biomol. Spectrosc.* 51(6), L7-L21. doi: 10.1016/0584-8539(95)01421-P.

Stavrov, S. S., Solntsev, K. M., Tolbert, L. M., and Huppert, D. (2006). Probing the decay coordinate of the green fluorescent protein: arrest of *cis−trans* isomerization by the protein significantly narrows the fluorescence spectra. *J. Am. Chem. Soc.* 128(5), 1540-1546. doi: 10.1021/ja0555421.

Tachibana, S. R., Tang, L., Zhu, L., Liu, W., Wang, Y., and Fang, C. (2018). Watching an engineered calcium biosensor glow: altered reaction pathways before emission. *J. Phys. Chem. B* 122(50), 11986–11995. doi: 10.1021/acs.jpcb.8b10587.

Tang, L., and Fang, C. (2019). Nitration of tyrosine channels photoenergy through a conical intersection in water. *J. Phys. Chem. B* 123(23), 4915-4928. doi: 10.1021/acs.jpcb.9b03464.

Tang, L., Liu, W., Wang, Y., Zhao, Y., Oscar, B. G., Campbell, R. E., et al. (2015). Unraveling ultrafast photoinduced proton transfer dynamics in a fluorescent protein biosensor for $Ca^{2+}$ imaging. *Chem. Eur. J.* 21(17), 6481-6490. doi: 10.1002/chem.201500491.

Tang, L., Liu, W., Wang, Y., Zhu, L., Han, F., and Fang, C. (2016). Ultrafast structural evolution and chromophore inhomogeneity inside a green-fluorescent-protein-based $Ca^{2+}$ biosensor. *J. Phys. Chem. Lett.* 7(7), 1225-1230. doi: 10.1021/acs.jpclett.6b00236.

Tang, L., Wang, Y., Liu, W., Zhao, Y., Campbell, R. E., and Fang, C. (2017). Illuminating photochemistry of an excitation ratiometric fluorescent protein calcium biosensor. *J. Phys. Chem. B* 121(14), 3016–3023. doi: 10.1021/acs.jpcb.7b01269.

Tang, L., Zhu, L., Taylor, M. A., Wang, Y., Remington, S. J., and Fang, C. (2018a). Excited state structural evolution of a GFP single-site mutant tracked by tunable femtosecond-stimulated Raman spectroscopy. *Molecules* 23(9)**,** 2226. doi: 10.3390/molecules23092226.

Tang, L., Zhu, L., Wang, Y., and Fang, C. (2018b). Uncovering the hidden excited state toward fluorescence of an intracellular pH indicator. *J. Phys. Chem. Lett.* 9(17)**,** 4969-4975. doi: 10.1021/acs.jpclett.8b02281.

Taylor, M. A., Zhu, L., Rozanov, N. D., Stout, K. T., Chen, C., and Fang, C. (2019). Delayed vibrational modulation of the solvated GFP chromophore into a conical intersection. *Phys. Chem. Chem. Phys.* 21(19)**,** 9728-9739. doi: 10.1039/C9CP01077G.

Tsien, R. Y. (1998). The green fluorescent protein. *Annu. Rev. Biochem.* 67(1)**,** 509-544. doi: 10.1146/annurev.biochem.67.1.509.

Wachter, R. M., Elsliger, M.-A., Kallio, K., Hanson, G. T., and Remington, S. J. (1998). Structural basis of spectral shifts in the yellow-emission variants of green fluorescent protein. *Structure* 6(10)**,** 1267-1277. doi: 10.1016/S0969-2126(98)00127-0.

Wang, L., Xie, J. M., Deniz, A. A., and Schultz, P. G. (2003). Unnatural amino acid mutagenesis of green fluorescent protein. *J. Org. Chem.* 68(1)**,** 174-176. doi: 10.1021/jo026570u.

Zhu, L., Liu, W., and Fang, C. (2014). A versatile femtosecond stimulated Raman spectroscopy setup with tunable pulses in the visible to near infrared. *Appl. Phys. Lett.* 105(4)**,** 041106. doi: 10.1063/1.4891766.
